# Supplementary material for: MetaSort untangles metagenome assembly by reducing microbial community complexity
Source: Nat Commun. 2017 Jan 23;8:14306. doi: 10.1038/ncomms14306 (PMC5264255; doi:10.1038/ncomms14306)
Supplement: Supplementary Information — Supplementary Figures 1-17, Supplementary Tables 1-10 [file ncomms14306-s1.pdf]

## The BAF Algorithm in metaSort

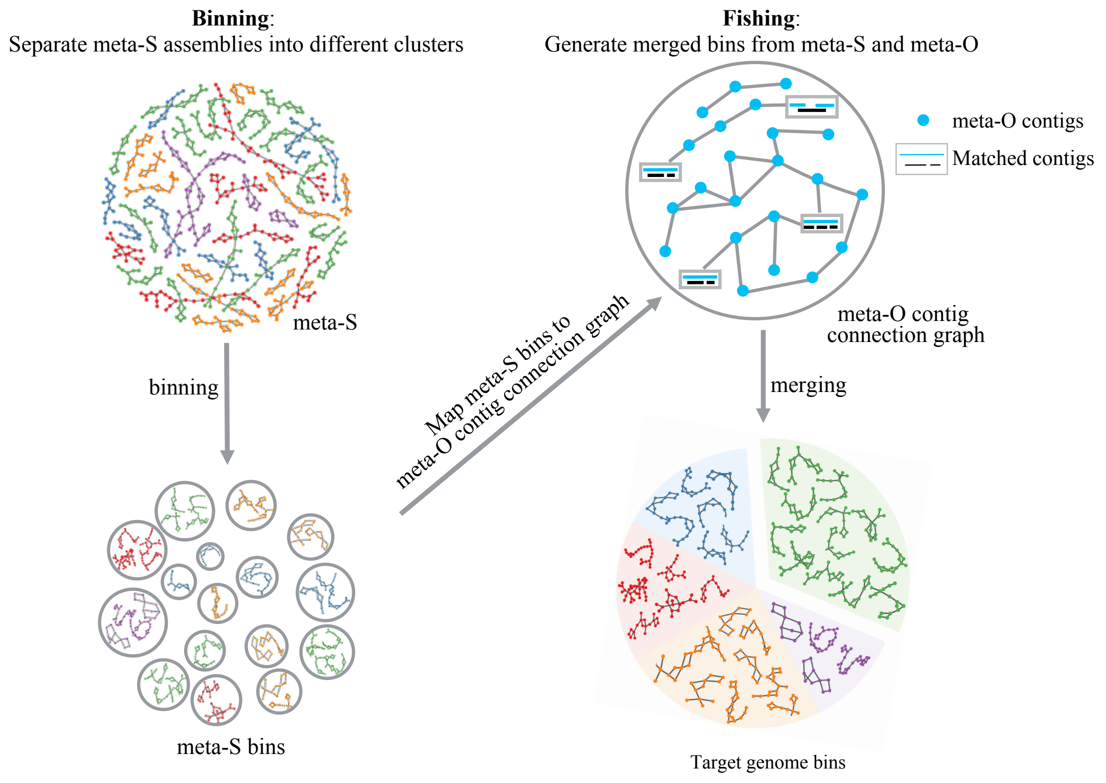


**Supplementary Figure 1. Overview of the BAF algorithm.** The SPAdes-assembled contigs are first clustered into meta-S bins by using the tetra-nucleotide frequency (TNF) features. Then, meta-S bins are mapped to the meta-O contig connection graph. For those meta-S bins locating on the meta-O contig connection graph, paths linking two bins are found. Subsequently, the meta-S bins and the paths linking meta-S bins are extracted to form a new graph. Finally, this graph is partitioned into separated components by graph connectivity and the meta-S bins in the same component are clustered into merged bins. The merged bins and the remainder of meta-S bins that do not participate in the process of merging constitute the target genome bins.

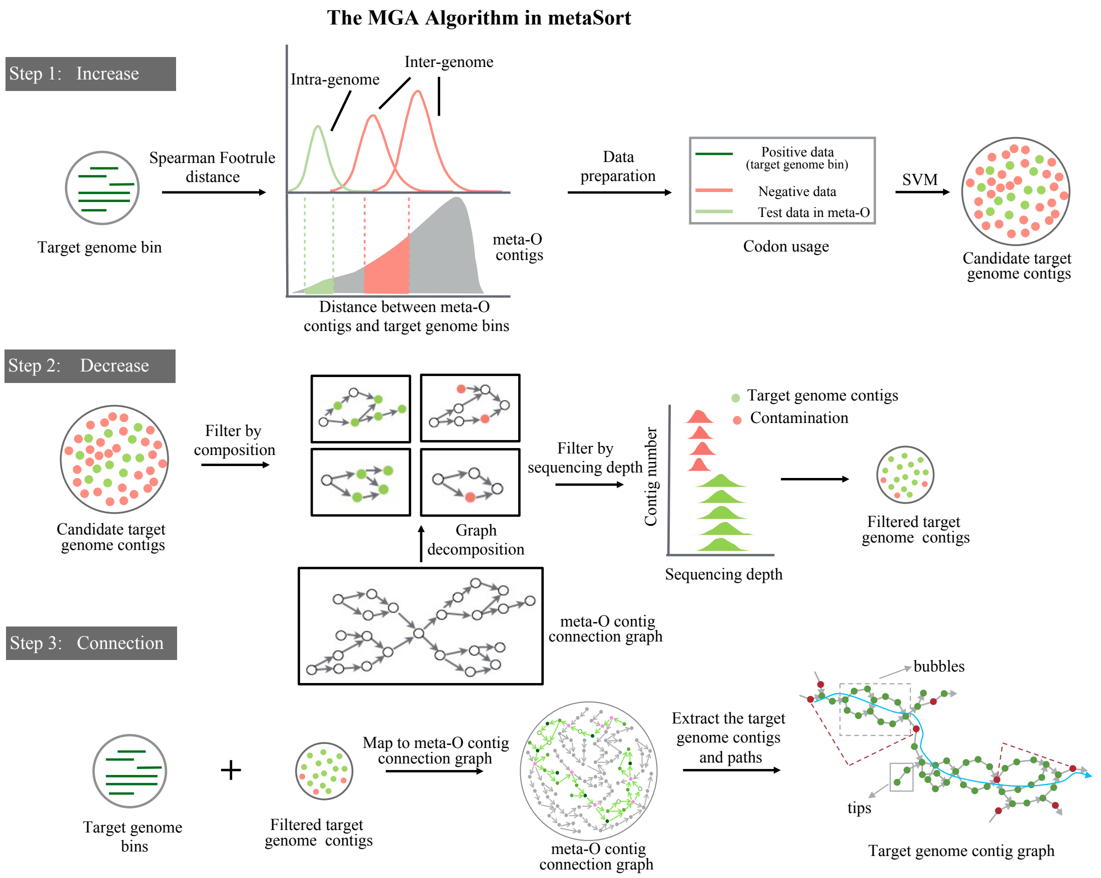


**Supplementary Figure 2. Overview of the MGA algorithm.** MGA is a three-step process that includes increase, decrease and connection steps. ***Step 1: Increase.*** Datasets are prepared for SVM classification using the Spearman Footrule distance. Meta-O contigs are compared with the target genome bin sequences to get the negative and test datasets. Then, SVM trains the classification model based on the positive and negative datasets using the codon usage as feature vector. Candidate target genome contigs are predicted by applying the classification model on the test dataset. ***Step 2: Decrease.*** The candidate target genome contigs are filtered by graph composition and sequencing depth. ***Step 3: Connection.*** Both the filtered target genome contigs and target genome bin sequences are mapped to the meta-O contig connection graph, and the matched contigs, referred to as landmarks, are used for traversing on the meta-O contig connection graph. MGA starts at each landmark and searches against the meta-O contig connection graph to obtain the paths connecting two landmarks. Once all the landmarks are visited on the meta-O contig connection graph, the landmarks along the searched paths are extracted to form a TGCG. Then

tips are removed, bubbles are merged and the remaining target genome contigs are assembled into scaffolds.

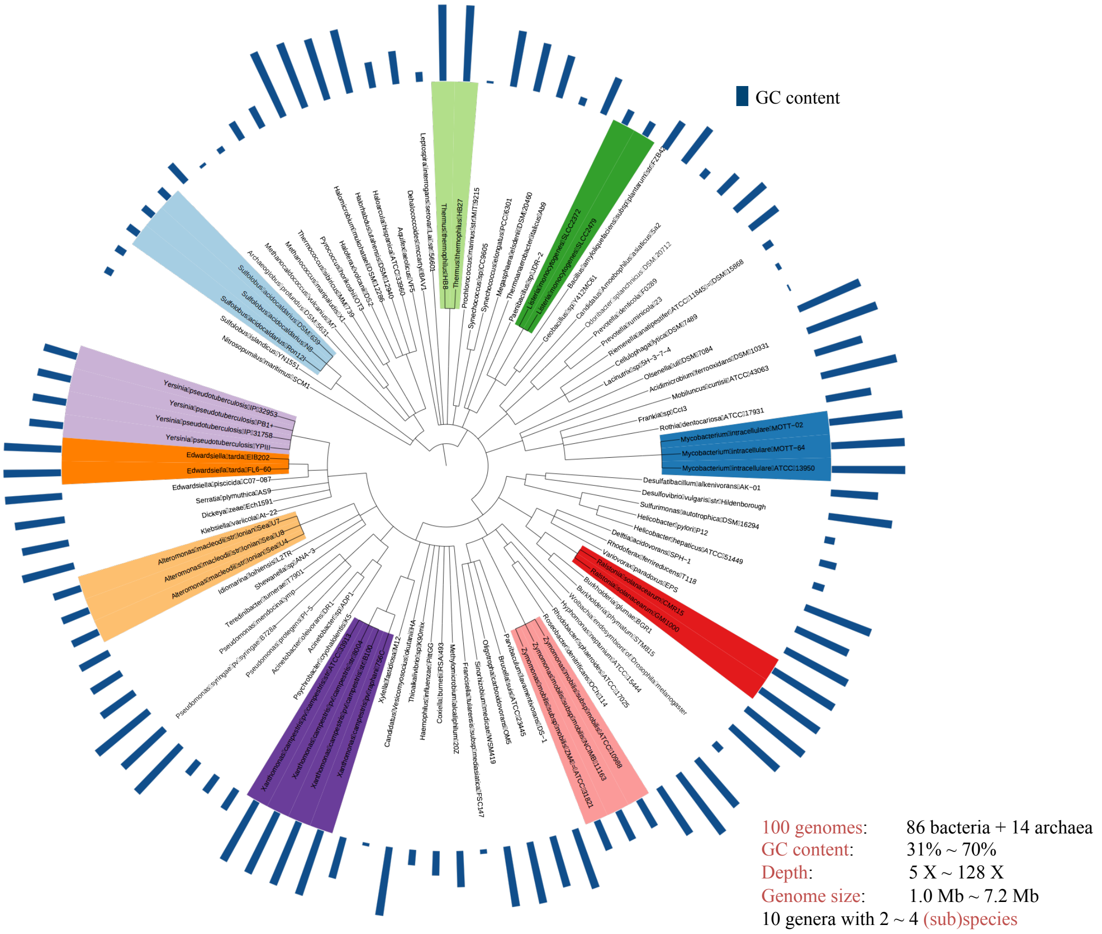


**Supplementary Figure 3. The simulated metagenomic dataset.** Phylogenetic tree of simulated bacterial genomes (inner), the taxonomic name of each genome (middle) and the GC content (outer). This metagneomic dataset contains 10 taxa that contain different number of subspecies or strains (highlighted in color).

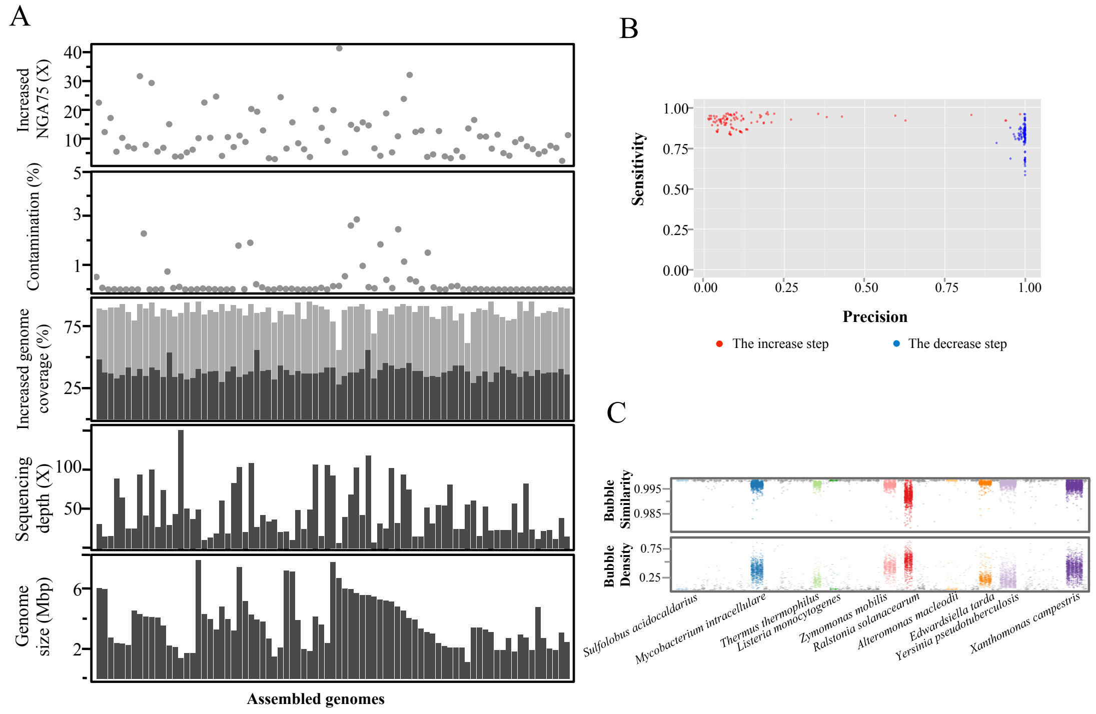


**Supplementary Figure 4. MGA assembly of the simulated metagenomic dataset.** (a) General statistics of the assembly results. (b) To demonstrate the efficiency of the decrease step in MGA, the contamination rate in the increase and decrease steps is estimated. Each point represents one genome. (c) Identification of strain-level variation based on bubble similarity and bubble density. The species containing different strains are represented by different colors.

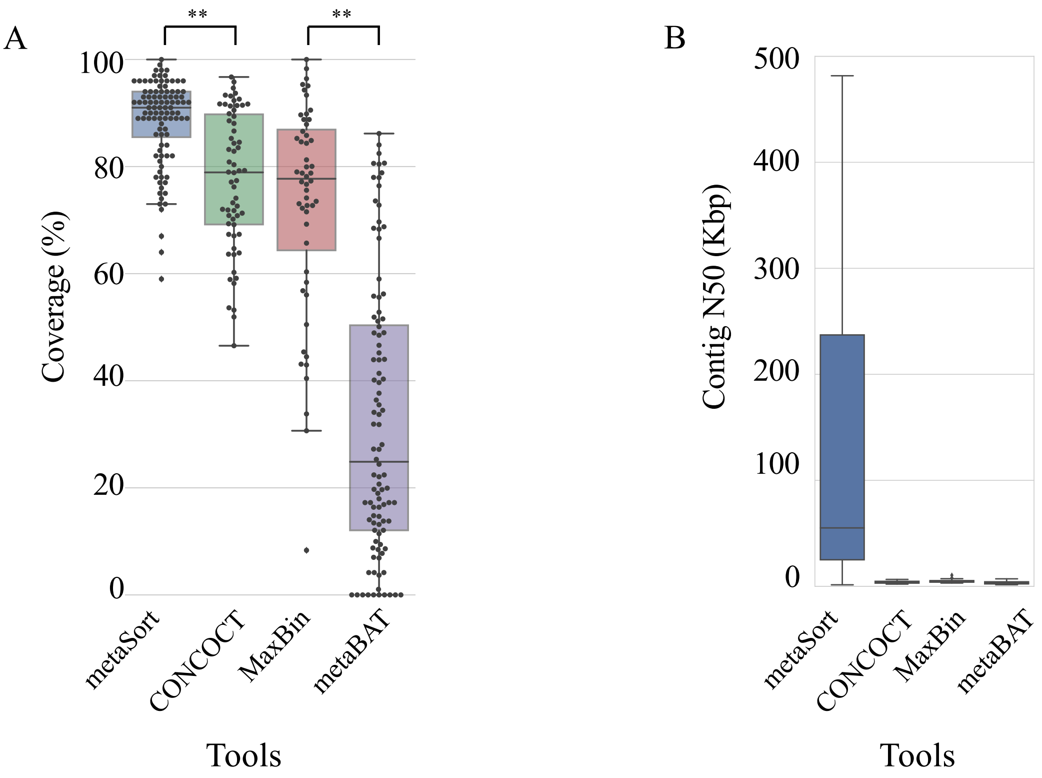


**Supplementary Figure 5. Performance comparison on genome coverage and contig N50 length among metaSort, CONCOCOT, MaxBin and metaBAT.** (a) The distribution of recovered genome coverage by each method. Each point represents one genome. *P* values were obtained using the *t*-test. (b) The distribution of contig N50 length by each method.

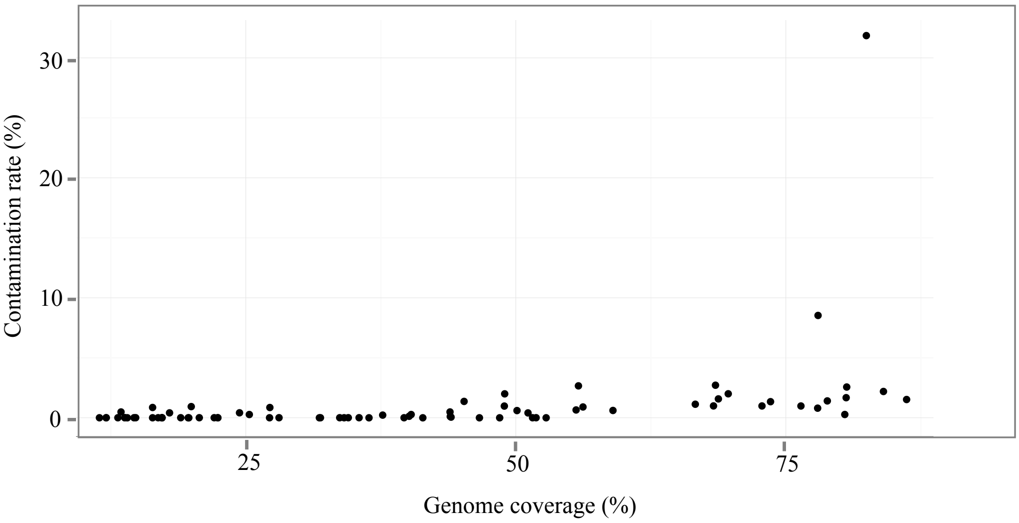


**Supplementary Figure 6. Performance the BAF algorithm on the meta-O assemblies**. X-axis indicates recovered genome coverage and y-axis presents contamination rate. Each point represents one genome.

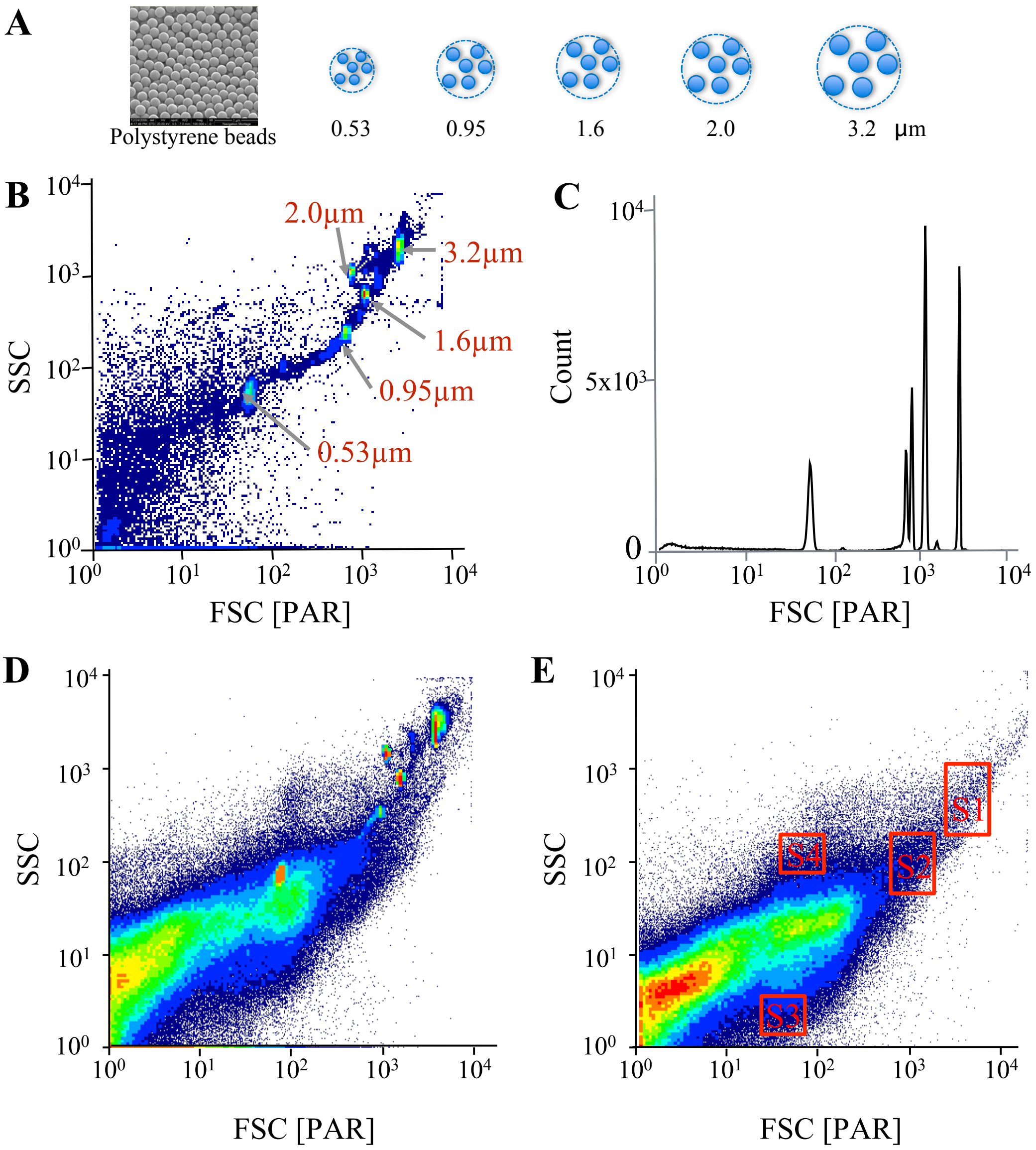


**Supplementary Figure 7. Segregation of subsets of microbial samples using FCM.** (a) The diameters of calibrated polystyrene beads used in our experiment. (b) The distribution of calibrated polystyrene beads in the dual-color fluorescence (FSC and SSC) dot plot. (c) Calibrated polystyrene beads with various nominal diameters are analyzed in FSC using a fluorescence-based discriminator. (d) FCM analysis of the oral microbial sample with polystyrene beads. (e) FCM analysis of the oral microbial sample without polystyrene beads. Four different gating windows (noted by red rectangle) are set to segregate the cells.

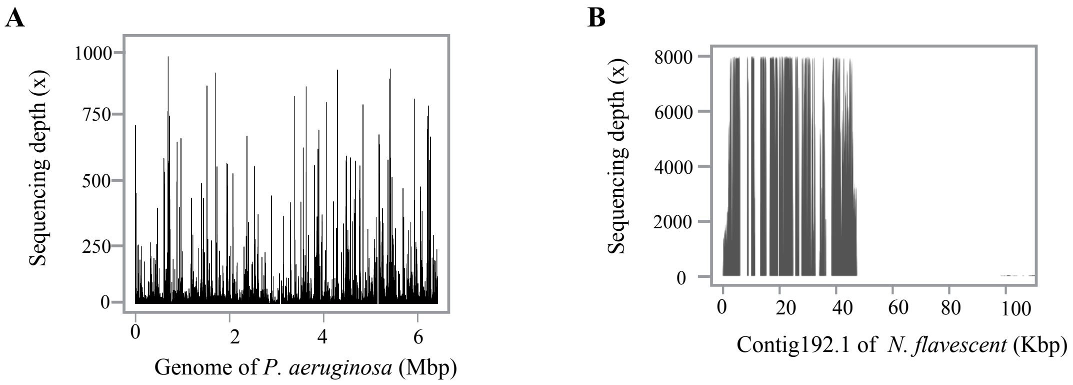


**Supplementary Figure 8. Illustration of MDA amplification bias.** Meta-S reads are mapped to the reference genomes and the sequencing depth of every base is calculated. (a) Distribution of sequencing depth along the *P. aeruginosa* genome. (b) Distribution of sequencing depth along the largest contig of *N. flavescent* genome.

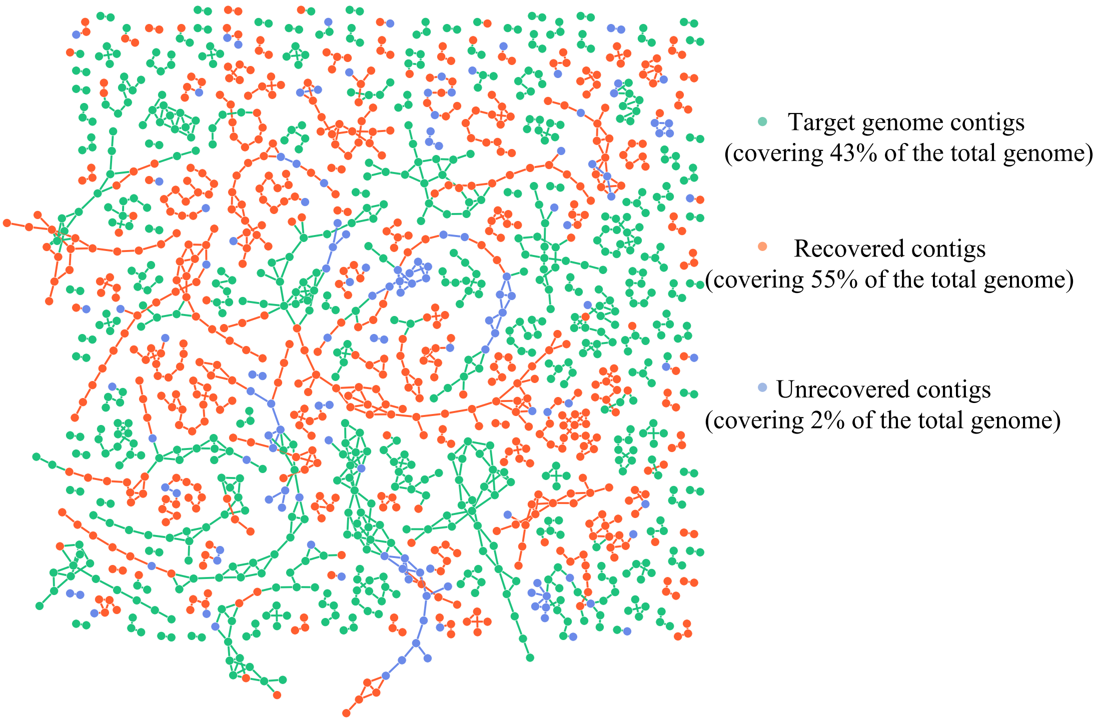


**Supplementary Figure 9. Contig connection graph of *P. aeruginosa.*** Each node denotes a contig and edge represents overlap between two contigs. The seed, recovered and unrecovered target contigs are colored with green, red, blue, respectively.

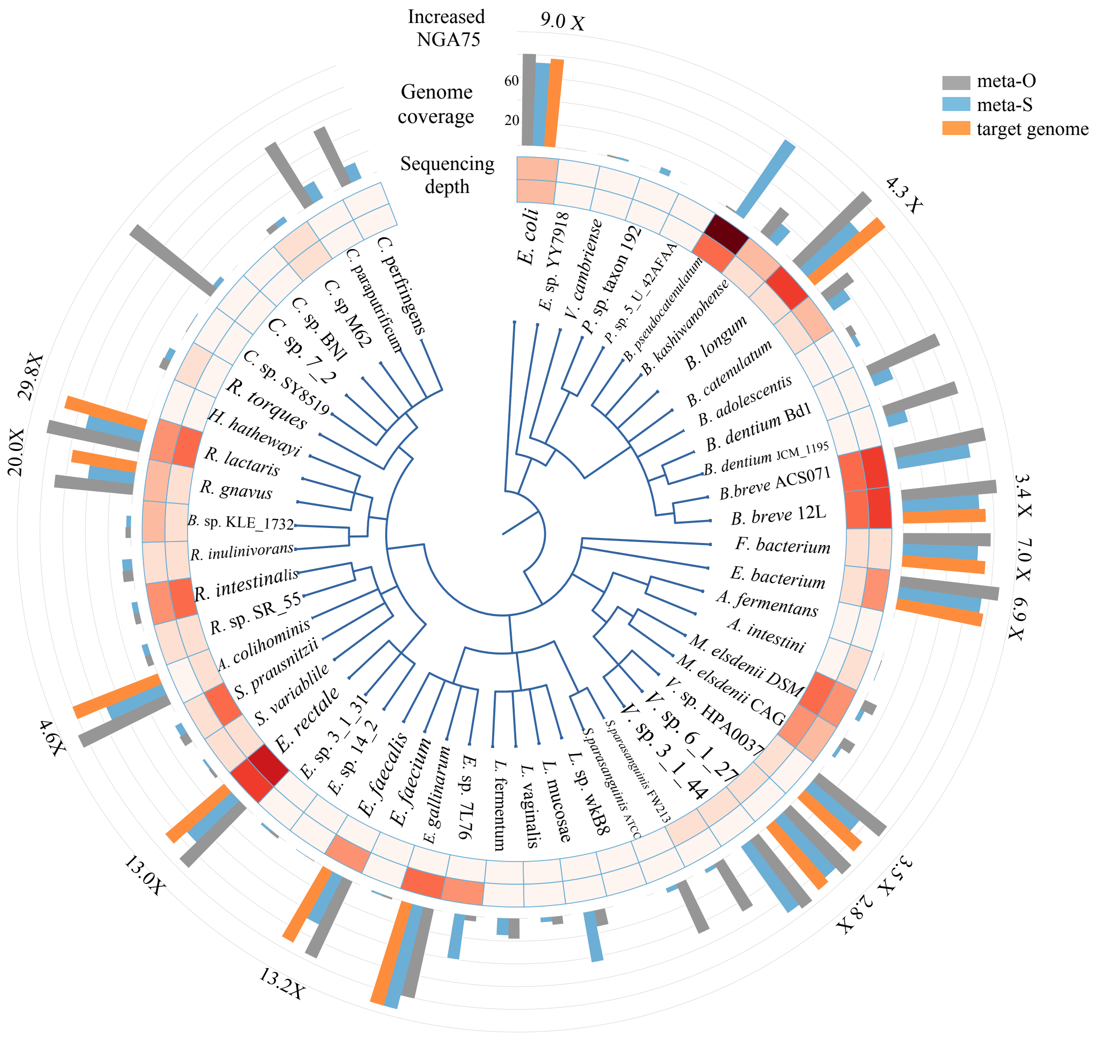


**Supplementary Figure 10. Application of metaSort on the gut metagenomic sample.** The heat map shows the normalized sequencing depth of each genome in the meta-O (inner) and meta-S (outer). Bar plot shows the genome coverage in the MGA assembly.

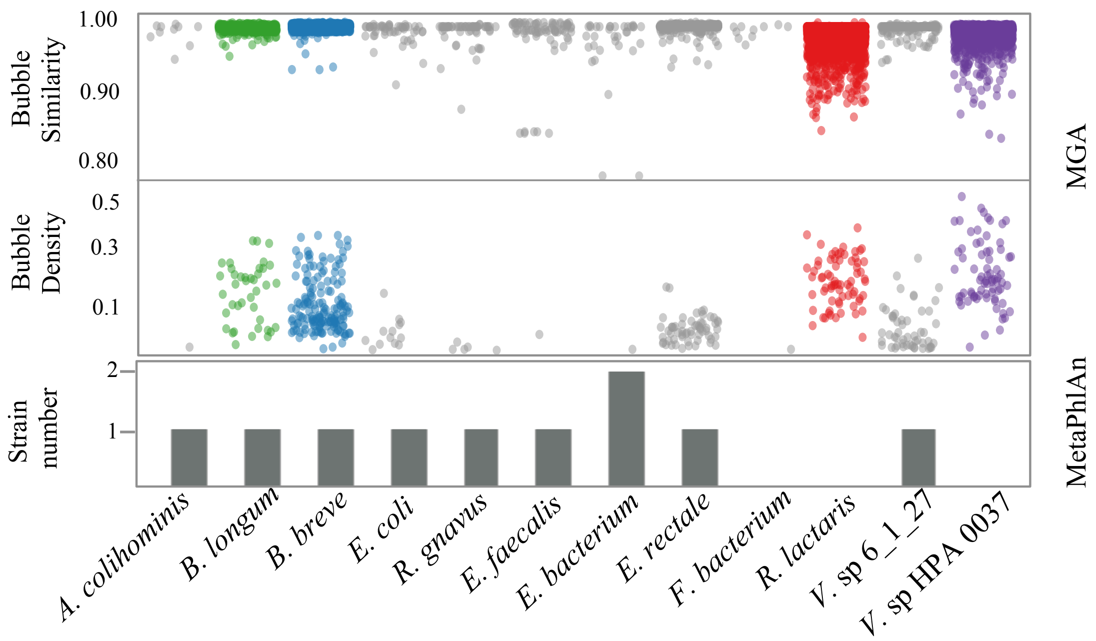


**Supplementary Figure 11. Comparison of variation detection in the gut metagenome between MGA and MetaPhlAn.** The bubble similarity and density of components in each assembled genome are represented by dot plot. The species that contain stain-level variations are colored in green, blue, red and purple, respectively. The predicted strain number of each species by MetaPhlAn is shown by bar plot.

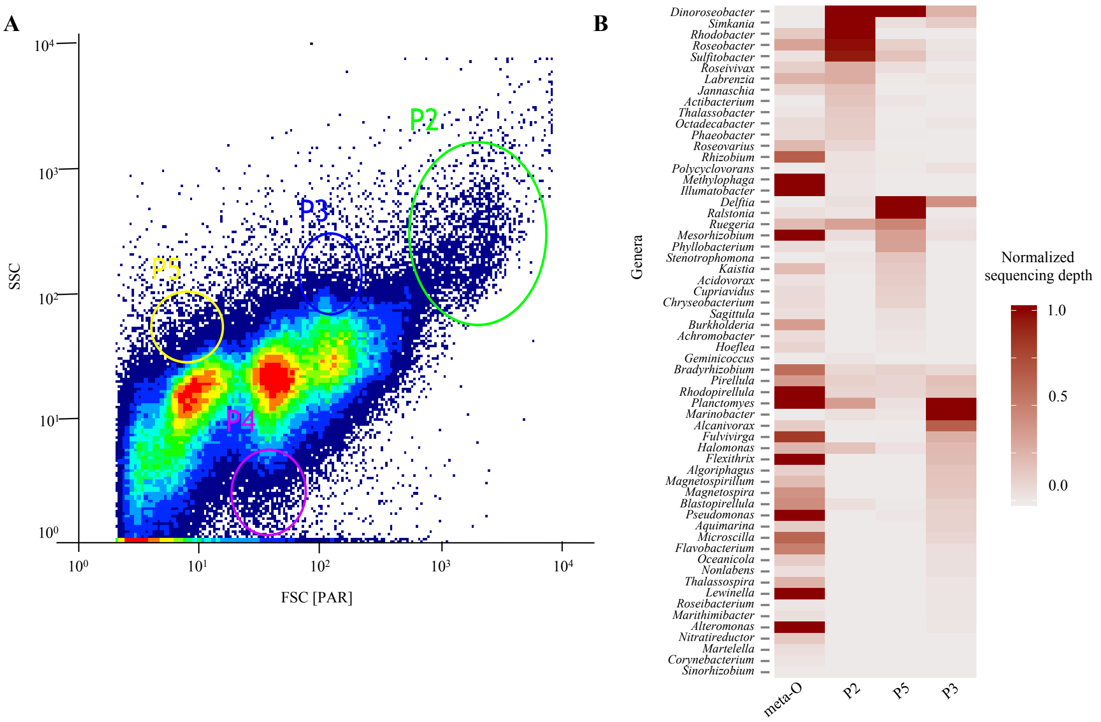


**Supplementary Figure 12. Application of metaSort on the kelp microbial sample.** (a) Gating windows for generating subsets of kelp microbial sample in the dual-color fluorescence plot. Four different regions are gated to recover the cells. (b) Heat map shows normalized abundance of enriched taxa in meta-O and three meta-S subsets. P4 was failed in MDA amplification and thus was abandoned.

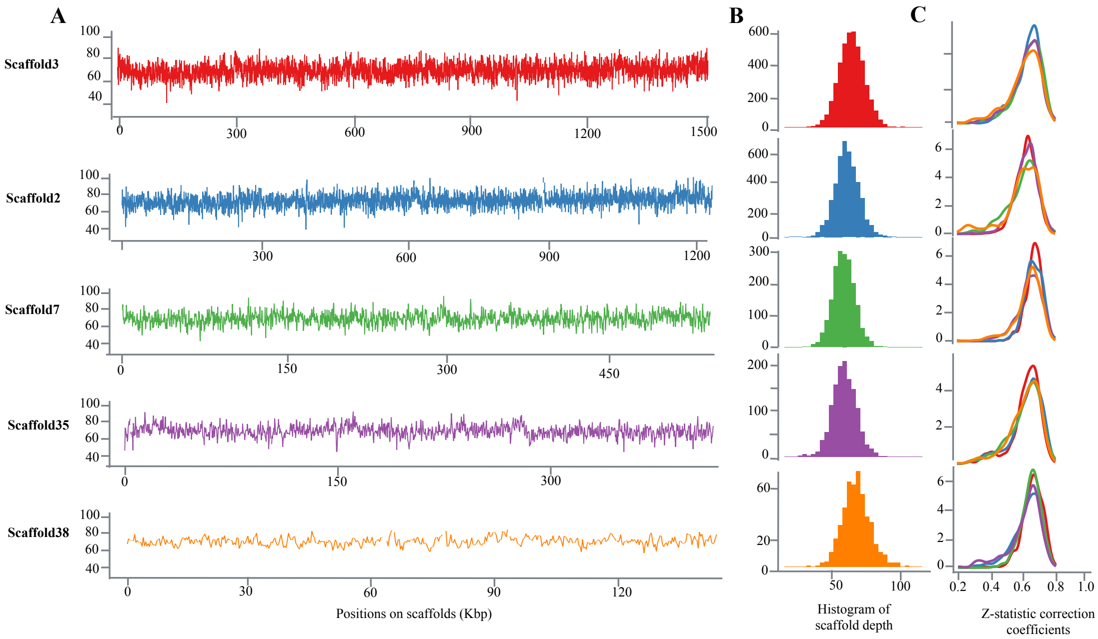


**Supplementary Figure 13. Validation of the HD560 assembly by sequencing depth and sequence composition.** The meta-O reads are mapped to the scaffolds of HD560 and then the sequencing depth of each scaffold is calculated. To illustrate the specificity of the assembly, the top five largest scaffolds are selected. The line plot (left) shows the distribution of sequencing depth on the scaffolds, with y-axis indicating the depth and x-axis representing the position on the scaffolds. The histogram (middle) illustrates the overall distribution of sequencing depth of the scaffolds (window size = 1 Kbp). Pairwise comparisons of these scaffolds based on the tetra-nucleotide Z-statistic correction coefficients are shown in density plot (right).

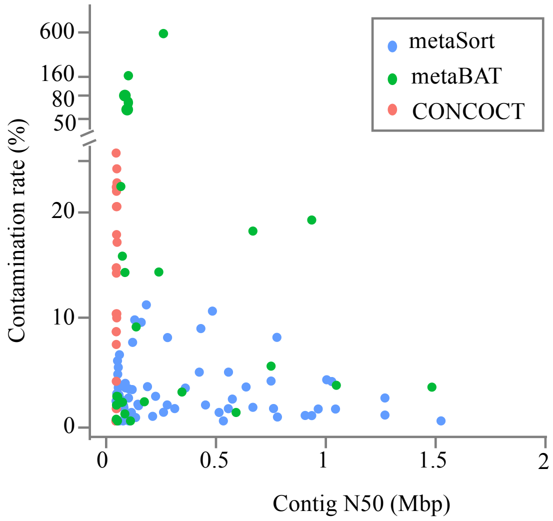


**Supplementary Figure 14. Comparison between metaSort, CONCOCT and metaBAT.** Each point represents one genome.

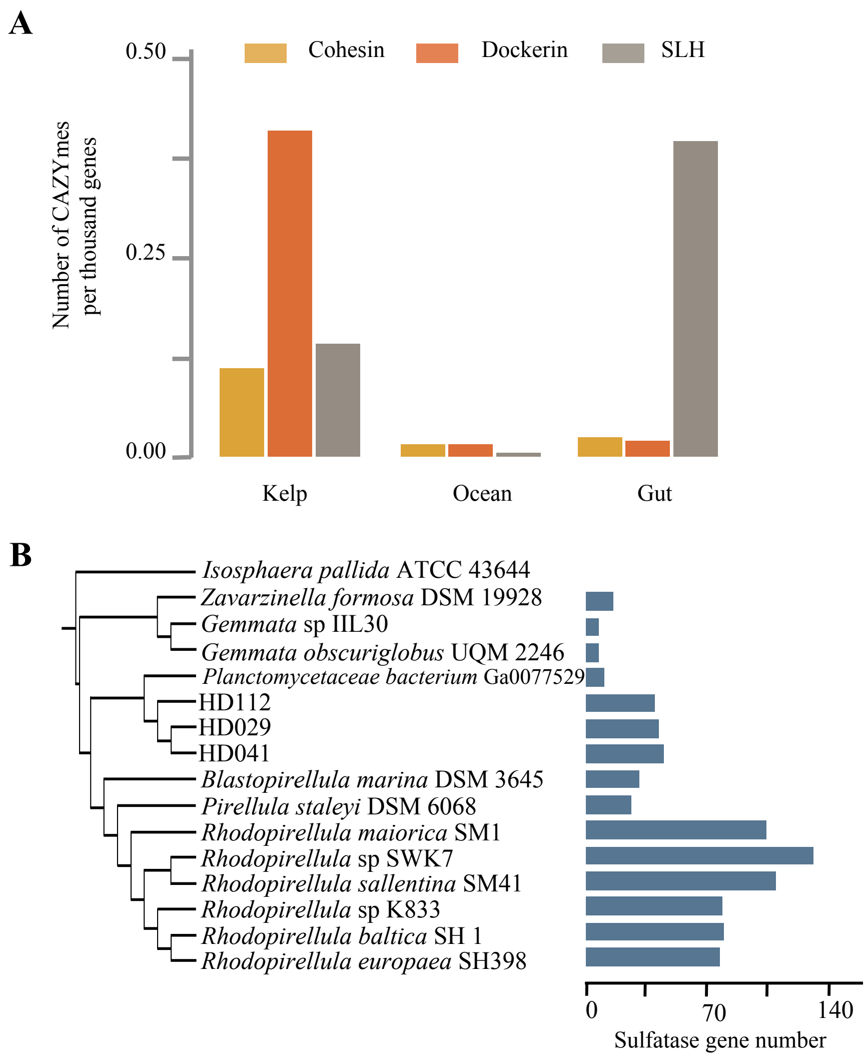


**Supplementary Figure 15. Cellulosome and sulfatase genes in the assembled kelp bacterial genomes.** (a) Percentage of cellulosome components in the assembled kelp bacterial genomes, gut and ocean microbiomes. (b) Abundance of sulfatase encoding genes in the Planctomycetaceae family. The sulfatase genes are annotated by scanning the genome with the EGGNOG 4.1 database.

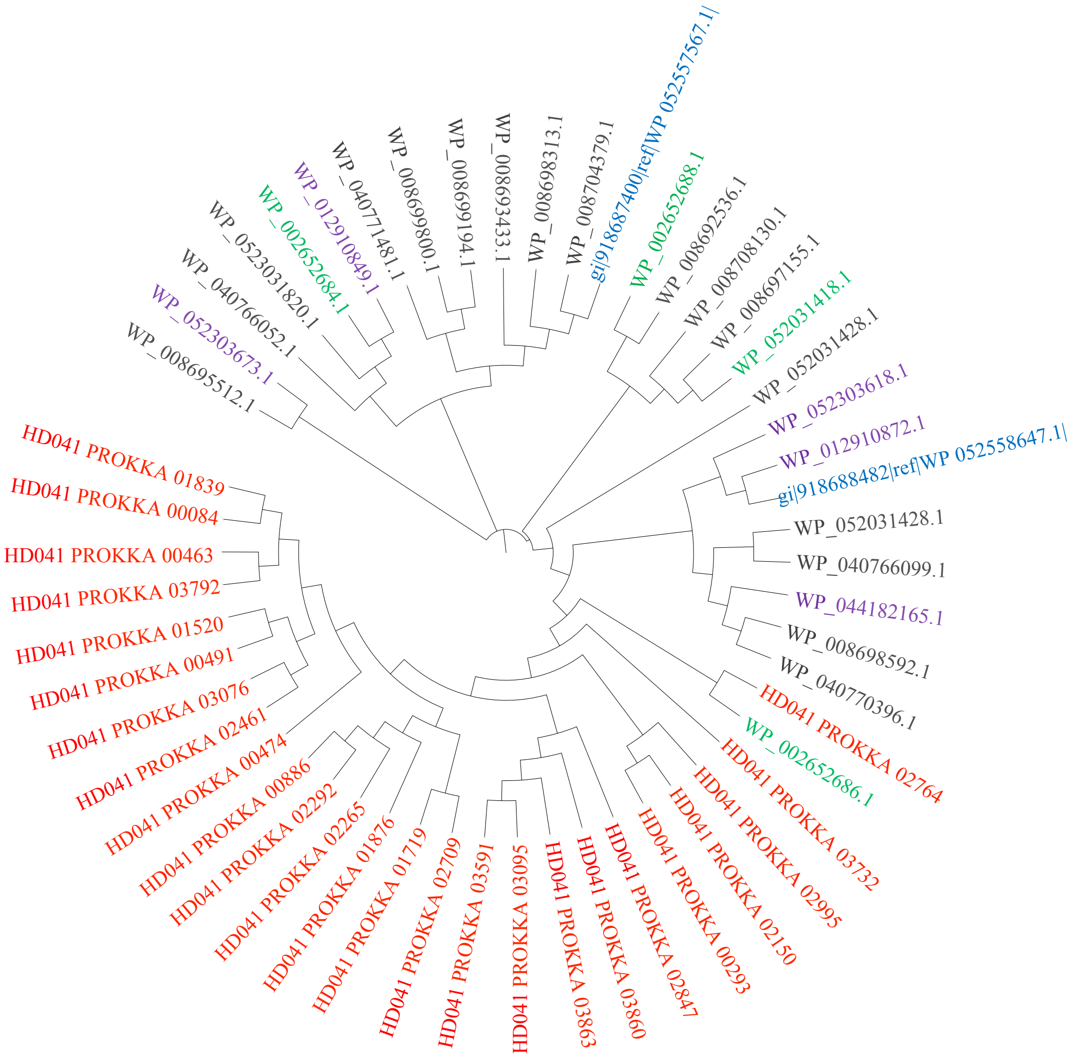


**Supplementary Figure 16. Phylogenetic tree of dockerin genes.** A phylogenetic tree is built based on the dockerin protein sequences in the genomes of HD041 (red), *Blastopirellula marina* DSM_3645 (green), *Pirellula staleyi* DSM_6068 (purple), *Rhodopirellula maiorica* SM1 (black) and *Gemmata* sp IIL30 (blue).

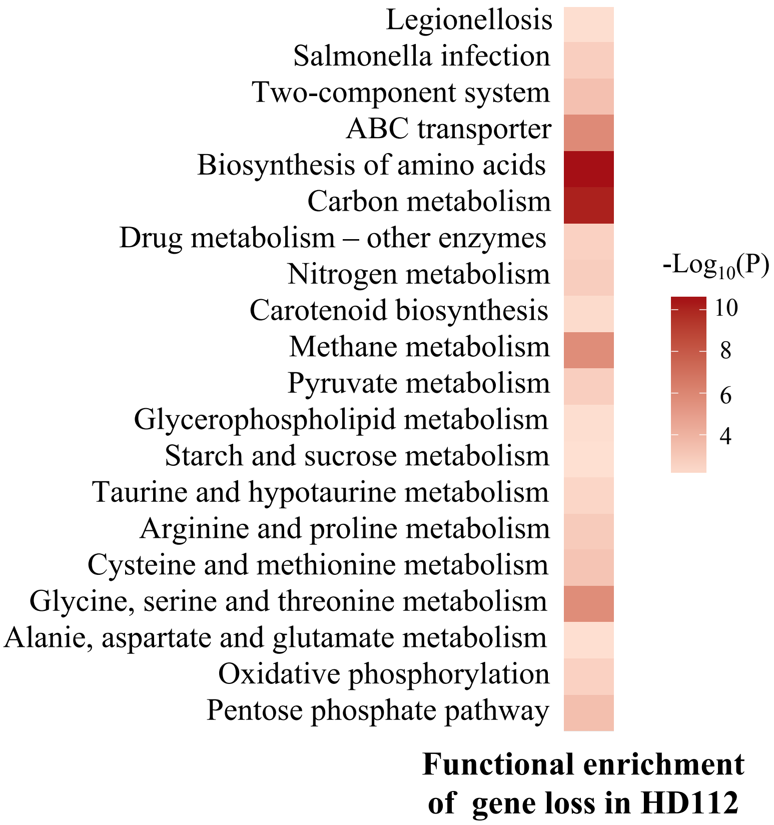


**Supplementary Figure 17. Functional enrichment of lost genes in HD112.** The heat map shows the enriched functions ($q < 0.01$, fisher exact and FDR test).

**Supplementary Table 1. General description of the simulated metagenomic dataset. The strain-containing species is highlighted by different colors.**

| Strain name | Taxonomy id | GC content (%) | Genome size | Sequencing depth (fold) |
|---|---|---|---|---|
| *Sulfolobus acidocaldarius* DSM 639_uid58379 | 330779 | 37 | 2,225,959 | 5 |
| *Sulfolobus acidocaldarius* N8_uid189027 | 1028566 | 37 | 2,176,362 | 14 |
| *Sulfolobus acidocaldarius* Ron12_I_uid189028 | 1028567 | 37 | 2,223,983 | 21 |
| *Sulfolobus islandicus* Y_N_15_51_ uid58825 | 419942 | 35 | 2,812,165 | 33 |
| *Archaeoglobus profundus* DSM 5631_uid43493 | 572546 | 42 | 1,560,622 | 32 |
| *Haloarcula hispanica* ATCC 33960_uid72475 | 634497 | 64 | 2,995,271 | 45 |
| *Haloferax volcanii* DS2 uid46845 | 309800 | 67 | 2,847,757 | 20 |
| *Halomicrobium mukohataei* DSM 12286_uid59107 | 485914 | 66 | 3,110,487 | 13 |
| *Halorhabdus utahensis* DSM 12940_uid59189 | 519442 | 63 | 3,116,795 | 52 |
| *Methanocaldococcus vulcanius* M7 uid41131 | 579137 | 31 | 1,746,329 | 20 |
| *Methanococcus maripaludis* X1_uid70729 | 1053692 | 33 | 1,746,697 | 20 |
| *Pyrococcus horikoshii* OT3 uid57753 | 70601 | 42 | 1,738,505 | 10 |
| *Thermococcus sibiricus* MM 739 uid59399 | 604354 | 40 | 1,845,800 | 20 |
| *Nitrosopumilus maritimus* SCM1 uid58903 | 436308 | 34 | 1,645,259 | 49 |
| *Acidimicrobium ferrooxidans* DSM 10331_uid59215 | 525909 | 68 | 2,158,157 | 75 |
| *Frankia* CcI3 uid58397 | 106370 | 70 | 5,433,628 | 13 |
| *Mobiluncus curtisii* ATCC 43063_uid49695 | 548479 | 55 | 2,146,480 | 55 |
| *Rothia dentocariosa* ATCC 17931_uid49331 | 762948 | 54 | 2,506,025 | 13 |
| *Mycobacterium* intracellulare ATCC 13950_uid167994 | 487521 | 68 | 5,402,402 | 22 |
| *Mycobacterium* intracellulare MOTT 02_uid89387 | 1138382 | 68 | 5,409,696 | 28 |
| *Mycobacterium* intracellulare MOTT 64_uid89385 | 1138383 | 68 | 5,501,090 | 30 |
| *Olsenella* uli DSM 7084_uid51367 | 633147 | 65 | 2,051,896 | 22 |
| *Aquifex aeolicus* VF5_uid57765 | 224324 | 43 | 1,551,335 | 43 |
| *Odoribacter splanchnicus* DSM 20712_uid63397 | 709991 | 43 | 4,392,288 | 52 |
| *Prevotella denticola* F0289 uid65091 | 767031 | 50 | 2,937,589 | 71 |
| *Prevotella ruminicola* 23_uid47507 | 264731 | 48 | 3,619,559 | 16 |
| *Cellulophaga lytica* DSM 7489_uid63401 | 867900 | 32 | 3,765,936 | 91 |
| *Lacinutrix* 5H 3_7_4_uid68067 | 983544 | 31 | 3,296,168 | 13 |
| *Riemerella anatipestifer* ATCC 11845_DSM_15868_uid60727 | 693978 | 35 | 2,155,121 | 90 |
| *Dehalococcoides* BAV1 uid58477 | 216389 | 47 | 1,341,892 | 31 |
| *Synechococcus elongatus* PCC 6301_uid58235 | 269084 | 55 | 2,696,255 | 17 |
| *Synechococcus* CC9605 uid58319 | 110662 | 59 | 2,510,659 | 70 |
| *Prochlorococcus marinus* MIT 9215_uid58819 | 93060 | 31 | 1,738,790 | 20 |

| | | | | |
|---|---|---|---|---|
| *Thermus thermophilus* HB8 uid58223 | 300852 | 70 | 1,849,742 | 25 |
| *Thermus thermophilus* HB27 uid58033 | 262724 | 69 | 1,894,877 | 11 |
| *Bacillus amyloliquefaciens* FZB42 uid58271 | 326423 | 46 | 3,918,589 | 9 |
| *Geobacillus* Y412MC61 uid41171 | 544556 | 52 | 3,622,844 | 12 |
| *Listeria monocytogenes* SLCC2372 uid174872 | 932920 | 38.0 | 2,972,810 | 34 |
| *Listeria monocytogenes* SLCC2479 uid175108 | 882020 | 38.0 | 2,972,172 | 16 |
| *Paenibacillus* JDR 2_uid59021 | 324057 | 50 | 7,184,930 | 42 |
| *Thermoanaerobacter italicus* Ab9 uid46241 | 580331 | 34 | 2,451,061 | 29 |
| *Megasphaera elsdenii* DSM 20460_uid71135 | 1064535 | 53 | 2,474,718 | 20 |
| *Oligotropha carboxidovorans* OM5 uid59155 | 504832 | 62 | 3,745,629 | 85 |
| *Brucella suis* ATCC 23445_uid59015 | 470137 | 57 | 1,923,763 | 37 |
| *Sinorhizobium medicae* WSM419 uid58549 | 366394 | 62 | 3,781,904 | 35 |
| *Parvibaculum lavamentivorans* DS 1_uid58739 | 402881 | 62 | 3,914,745 | 80 |
| *Hyphomonas neptunium* ATCC 15444_uid58433 | 228405 | 62 | 3,705,021 | 23 |
| *Rhodobacter sphaeroides* ATCC 17025_uid58451 | 349102 | 68 | 3,217,726 | 63 |
| *Roseobacter denitrificans* OCh 114_uid58597 | 375451 | 59 | 4,133,097 | 21 |
| *Wolbachia endosymbiont* of Drosophila melanogaster uid57851 | 163164 | 35 | 1,267,782 | 128 |
| *Zymomonas mobilis* ATCC 10988_uid55403 | 555217 | 46 | 2,021,773 | 25 |
| *Zymomonas mobilis* NCIMB 11163_uid41019 | 622759 | 47 | 2,124,771 | 11 |
| *Zymomonas mobilis* ZM4 uid58095 | 264203 | 46 | 2,056,363 | 26 |
| *Burkholderia glumae* BGR1 uid59397 | 626418 | 68 | 3,906,507 | 92 |
| *Burkholderia phymatum* STM815 uid58699 | 391038 | 63 | 3,479,187 | 36 |
| *Ralstonia solanacearum* GMI1000 uid57593 | 267608 | 67.0 | 3,716,413 | 20 |
| *Ralstonia solanacearum* CMR15 uid227773 | 859655 | 67 | 3,596,030 | 23 |
| *Delftia acidovorans* SPH 1_uid58703 | 398578 | 66 | 6,767,514 | 88 |
| *Rhodoferax ferrireducens* T118 uid58353 | 338969 | 60 | 4,712,337 | 18 |
| *Variovorax paradoxus* EPS uid62107 | 595537 | 66 | 6,550,056 | 18 |
| *Desulfatibacillum alkenivorans* AK 01 uid58913 | 439235 | 54 | 6,517,073 | 9 |
| *Desulfovibrio vulgaris* Hildenborough uid57645 | 882 | 63 | 3,570,858 | 41 |
| *Helicobacter hepaticus* ATCC 51449 uid57737 | 235279 | 36 | 1,799,146 | 21 |
| *Helicobacter pylori* P12 uid59327 | 570508 | 39 | 1,673,813 | 42 |
| *Sulfurimonas autotrophica* DSM 16294_uid53043 | 563040 | 35 | 2,153,198 | 47 |
| *Alteromonas macleodii* Ionian Sea U4_uid210780 | 1300255 | 45 | 4,321,296 | 12 |
| *Alteromonas macleodii* Ionian Sea U8_uid210782 | 1300257 | 45 | 4,395,035 | 28 |
| *Alteromonas macleodii* Ionian Sea U7_uid210785 | 1300256 | 45 | 4,442,936 | 14 |
| *Teredinibacter turnerae* T7901_uid59267 | 377629 | 51 | 5,193,164 | 88 |
| *Idiomarina loihiensis* L2TR uid58087 | 283942 | 47 | 2,839,318 | 44 |
| *Shewanella* ANA 3_uid58347 | 94122 | 48 | 4,972,204 | 7 |

| | | | | |
|---|---|---|---|---|
| *Thioalkalivibrio* K90mix uid46181 | 396595 | 66 | 2,744,800 | 27 |
| *Dickeya zeae* Ech1591 uid59297 | 561229 | 55 | 4,813,854 | 27 |
| *Edwardsiella piscicida* C07 087_uid193773 | 1288122 | 60 | 3,857,040 | 21 |
| *Edwardsiella tarda* EIB202 uid41819 | 498217 | 60 | 3,760,463 | 29 |
| *Edwardsiella tarda* FL6 60_uid159657 | 718251 | 60 | 3,684,607 | 64 |
| *Klebsiella variicola* At 22_uid42113 | 640131 | 58 | 5,458,505 | 34 |
| *Serratia plymuthica* AS9 uid67313 | 768492 | 56 | 5,442,880 | 58 |
| *Yersinia pseudotuberculosis* PB1 uid59153 | 502801 | 48 | 4,695,619 | 35 |
| *Yersinia pseudotuberculosis* IP 31758_uid58487 | 349747 | 48 | 4,723,306 | 11 |
| *Yersinia pseudotuberculosis* IP 32953_uid58157 | 273123 | 48 | 4,744,671 | 22 |
| *Yersinia pseudotuberculosis* YPIII uid59151 | 502800 | 48 | 4,689,441 | 9 |
| *Coxiella burnetii* RSA 493_uid57631 | 227377 | 43 | 1,995,281 | 46 |
| *Methylomicrobium alcaliphilum* uid77119 | 1091494 | 49 | 4,668,296 | 87 |
| *Haemophilus influenzae* PittGG uid58593 | 374931 | 38 | 1,887,192 | 21 |
| *Acinetobacter* ADP1 uid61597 | 62977 | 40 | 3,598,621 | 13 |
| *Acinetobacter oleivorans* DR1 uid50119 | 436717 | 39 | 4,152,543 | 80 |
| *Psychrobacter cryohalolentis* K5 uid58373 | 335284 | 42 | 3,059,876 | 13 |
| *Pseudomonas fluorescens* Pf 5 uid57937 | 220664 | 63 | 7,074,893 | 79 |
| *Pseudomonas mendocina* ymp uid58723 | 399739 | 65 | 5,072,807 | 100 |
| *Pseudomonas syringae* B728a uid57931 | 205918 | 59 | 6,093,698 | 6 |
| *Candidatus Amoebophilus* asiaticus 5a2_uid58963 | 452471 | 35 | 1,884,364 | 21 |
| *Candidatus Vesicomyosocius* okutanii HA_uid59427 | 412965 | 32 | 1,022,154 | 48 |
| *Francisella tularensis* mediasiatica FSC147_uid58939 | 441952 | 32 | 1,893,886 | 69 |
| *Xanthomonas campestris* raphani 756C_uid159539 | 990315 | 65 | 4,941,214 | 10 |
| *Xanthomonas campestris* 8004_uid57595 | 314565 | 65.0 | 5,148,708 | 21 |
| *Xanthomonas campestris* ATCC 33913_uid57887 | 190485 | 65 | 5,076,188 | 36 |
| *Xanthomonas campestris* uid61643 | 509169 | 65.0 | 5,079,002 | 22 |
| *Xylella fastidiosa* M12_uid58763 | 405440 | 52 | 2,475,130 | 19 |
| *Leptospira interrogans* serovar Lai 56601_uid57881 | 189518 | 35 | 4,338,762 | 10 |

**Supplementary Table 2. MGA assembly results of genomes enriched in the meta-S of the oral sample.**

| | | *Actinomyces* sp. ICM47 | *Enterobacter cloacae* | *Prevotella pallens* | *Prevotella histicola* | *Prevotella salivae* | *Tannerella* sp. oral taxon BU063 | *Pseudomonas aeruginosa* |
|---|---|---|---|---|---|---|---|---|
| metaSort | **TGB contigs** | | | | | | | |
| | Number | 258 | 84 | 226 | 216 | 646 | 90 | 375 |
| | Size (bp) | 829,978 | 4,948,677 | 1,666,238 | 1,677,485 | 1,294,865 | 1,442,169 | 2,900,171 |
| | Genome coverage (%) | 33 | 88.16 | 52 | 54 | 36 | 35 | 44 |
| | **Recovered TG contigs** | | | | | | | |
| | Number | 2,780 | 11,806 | 3,156 | 2,923 | 10,983 | 1,094 | 1,858 |
| | Size (bp) | 2,416,325 | 3,710,841 | 2,965,719 | 3,007,517 | 4,922,484 | 2,471,194 | 6,484,357 |
| | NGA50 (bp) | 2,393 | 203 | 9,103 | 16,932 | 612 | 462 | 8,197 |
| | NGA75 (bp) | 71 | 71 | 387 | 2,628 | 221 | 71 | 3,449 |
| | **MGA scaffolds** | | | | | | | |
| | Number | 997 | 421 | 1,231 | 2,280 | 6,738 | 400 | 1,313 |
| | Size (bp) | 2,674,844 | 5,019,562 | 3,434,412 | 4,663,587 | 5,888,962 | 3,028,863 | 7,215,249 |
| | Genome coverage (%) | 70 | 88 | 76 | 81 | 67 | 52 | 88 |
| | NGA50 (bp) | 7,191 | 96,243 | 20,252 | 36,279 | 11,997 | 6,183 | 15,780 |
| | NGA75 (bp) | 2,926 | 35,539 | 10,757 | 26,928 | 4,773 | NA | 7,909 |
| | | | | | | | | |
| | Increased NGA50 (%) | 3.01 | 474.10 | 2.22 | 2.14 | 19.60 | 13.38 | 1.93 |
| | Increased NGA75 (%) | 41.21 | 500.55 | 27.80 | 10.25 | 21.60 | 13.38 | 2.29 |
| | Meta-O coverage (%) | 89.13 | 61.79 | 86.46 | 86.34 | 81.87 | 58.65 | 90.51 |
| | Enhanced genome coverage (%) | 36.2 | 0.0 | 23.7 | 26.5 | 30.7 | 17.3 | 44.4 |
| | Contamination | 15.4% | 8.8% | 4.9% | 6.9% | 2.5% | 4.4% | 7.5% |
| | | | | | | | | |
| metaBAT 1 | Genome coverage (%) | NA | NA | 56.407 | 65.534 | NA | NA | 80.148 |
| | NGA50 (bp) | NA | NA | 5,946 | 9,932 | NA | NA | 8,073 |
| | NGA75 (bp) | NA | NA | NA | NA | NA | NA | 3,404 |
| | | | | | | | | |
| metaBAT 2 | Genome coverage (%) | 66.81 | 89.20 | 51.509 | 60.82 | 15.57 | 44.42 | 78.127 |
| | NGA50 (bp) | 5,229 | 96,243 | 17,509 | 25,301 | NA | NA | 8,828 |
| | NGA75 (bp) | 3,134 | 38,014 | 8,837 | 17,863 | NA | NA | 4,099 |
| | Contamination | 69.4% | 9.2% | 3.5% | 71.3% | 5.2% | 16.2% | 6.7% |
| | | | | | | | | |
| CONCOCT2 | Genome coverage (%) | NA | 89.24 | NA | 78.89 | 25.4 | 54.58 | 78.33 |
| | NGA50 (bp) | NA | 96,243 | NA | 29,270 | NA | 3018 | 8,828 |
| | NGA75 (bp) | NA | 41,136 | NA | 21,435 | NA | NA | 4,109 |
| | Contamination | NA | 5.5% | NA | 71.4% | 79.3% | 18.2% | 14.5% |

metaBAT 1: performing metaBAT on meta-O assembly; metaBAT 2: performing metaBAT on merged sequences of meta-O and meta-S assemblies; CONCOCT 2: performing CONCOCT on merged sequences of meta-O and meta-S assemblies;

**Supplementary Table 3. General description of the recovered genome with initial genome coverage between 10% to 30%**

| | Initial | | | MGA | | | |
|---|---|---|---|---|---|---|---|
| Genome | Genome coverage (%) | Mean length (bp) | Maximum length (bp) | Genome coverage (%) | Mean length (bp) | Maximum length (bp) | Contamination rate (%) |
| bin.17 | 30 | 9,664 | 31,446 | 70 | 5,316 | 49,131 | 8.37 |
| bin.2 | 29 | 21,535 | 138,199 | 82 | 25,372 | 144,338 | 9.66 |
| bin.10 | 24 | 8,602 | 18,801 | 81 | 10,730 | 60,642 | 10.91 |
| bin.18 | 21 | 16,238 | 64,912 | 84 | 19,523 | 111,854 | 17.47 |
| bin.7 | 19 | 11,126 | 30,524 | 78 | 4,373 | 49,885 | 26.42 |

**Supplementary Table 4. Application of metaSort, metaSPAdes and IDBA-UD on genomes enriched in the meta-S of the oral sample.**

| | | *Actinomyces* sp. ICM47 | *Enterobacter cloacae* | *Prevotella pallens* | *Prevotella histicola* | *Prevotella salivae* | *Tannerella* sp. oral taxon BU063 | *Pseudomonas aeruginosa* |
|---|---|---|---|---|---|---|---|---|
| NGA50 | SOAPdenovo | 3,386 | NA | 10,901 | 17,389 | 958 | 2,407 | 8,251 |
| | IDBA-UD | 3,302 | 199 | 13,437 | 20,064 | 3,335 | 3,360 | 7,653 |
| | metaSPAdes | 9,308 | 509 | 19,803 | 27,569 | 19,288 | 3,172 | 15,263 |
| | metaSort 1 | 7,191 | 96,243 | 20,252 | 36,279 | 11,997 | 6,183 | 15,780 |
| | metaSort 2 | 9,219 | 96,243 | 22,793 | 29,063 | 30,005 | 8,598 | 25,844 |
| | | | | | | | | |
| NGA75 | SOAPdenovo | 848 | NA | 1,950 | 4,452 | 370 | NA | 3,577 |
| | IDBA-UD | 957 | NA | 3,080 | 4,477 | 747 | NA | 3,244 |
| | metaSPAdes | 3,506 | 208 | 4,692 | 11,063 | 4,549 | NA | 7,039 |
| | metaSort 1 | 2,926 | 35,539 | 10,757 | 26,928 | 4,773 | NA | 7,909 |
| | metaSort 2 | 4,647 | 35,539 | 14,383 | 18,725 | 17,868 | NA | 13,791 |
| | | | | | | | | |
| N's per 100 kbp | SOAPdenovo | 88 | 91 | 68 | 68 | 68 | 68 | 68 |
| | IDBA-UD | 0 | 0 | 0 | 0 | 0 | 0 | 0 |
| | metaSPAdes | 255 | 255 | 255 | 359 | 255 | 255 | 255 |
| | metaSort 1 | 4 | 3 | 2 | 0 | 9 | 1 | 9 |
| | metaSort 2 | 23 | 3 | 255 | 359 | 16 | 59 | 32 |
| | | | | | | | | |
| Misassemblies | SOAPdenovo | 74 | 7 | 117 | 28 | 154 | 295 | 34 |
| | IDBA-UD | 71 | 12 | 111 | 29 | 86 | 340 | 68 |
| | metaSPAdes | 104 | 21 | 124 | 38 | 112 | 398 | 47 |
| | metaSort 1 | 82 | 17 | 120 | 44 | 123 | 443 | 43 |
| | metaSort 2 | 108 | 17 | 127 | 46 | 131 | 454 | 61 |

metaSort 1: performing MGA based on SOAPdenovo assembly result; metaSort 2: performing MGA based on metaSPAdes assembly result. The most continuous two assemblies were highlighted.

**Supplementary Table 5. ANI between three assembled species of *Prevotella* and the genomes in the same genus in the oral reference database.**

| | Reference genomes | | | | | |
|---|---|---|---|---|---|---|
| Assembled genomes | *P. histicola* | *P. pallens* | *P. salivae* | *P. tannerae* | *P. veroralias* | *P.* sp. oral taxon 473 |
| *P. histicola* | 97.91 | 71.1 | 68.97 | 65.27 | 76.42 | 65.02 |
| *P. pallens* | 72.09 | 98.29 | 68.27 | 65.65 | 70.23 | 64.45 |
| *P. salivae* | 75.06 | 73.95 | 97.06 | 69.41 | 75.11 | 66.64 |

## Supplementary Table 6. MGA assembly results of genomes enriched in the meta-S of the gut sample.

| | | *Anaerotruncus colihominis* | *Enterococcus faecalis* | *Escherichia coli* | *Firmicutes bacterium* | *Erysipelotrichaceae bacterium* | *Bifidobacterium longum* | *Bifidobacterium breve* | *Eubacterium rectale* | *Ruminococcus lactaris* | *Ruminococcus gnavus* | *Veillonella* sp. HPA 0037 | *Veillonella* sp. 6_1_27 |
|---|---|---|---|---|---|---|---|---|---|---|---|---|---|
| metaSort | **Contigs in target genome bin** | | | | | | | | | | | | |
| | Number | 1,124 | 12 | 175 | 13 | 87 | 1,752 | 2,174 | 2,504 | 1,574 | 878 | 1,888 | 3,562 |
| | Size (bp) | 2,150,701 | 1,643,928 | 4,261,005 | 1,412,272 | 4,104,215 | 1,447,732 | 1,631,777 | 1,942,320 | 1,625,960 | 1,331,913 | 1,221,998 | 1,703,638 |
| | Genome coverage (%) | 55.5 | 47.3 | 76.4 | 40.9 | 78.5 | 60.8 | 67.9 | 53.0 | 50.9 | 41.1 | 54.5 | 68.0 |
| | NGA50 (bp) | 538 | 51 | 21,387 | 51 | 38,089 | 619 | 901 | 312 | 247 | 51 | 325 | 462 |
| | NGA75 (bp) | 51 | 51 | 1,693 | 51 | 9,136 | 51 | 51 | 51 | 51 | 51 | 75 | 159 |
| | **Recovered target genome contigs** | | | | | | | | | | | | |
| | Number | 301 | 316 | 1,086 | 404 | 672 | 18,774 | 119,231 | 16,041 | 4,970 | 1,928 | 19,543 | 16,608 |
| | Size (bp) | 3,249,298 | 2,443,282 | 4,785,642 | 2,820,323 | 4,493,686 | 4,393,092 | 16,637,947 | 4,637,782 | 3,192,562 | 2,074,692 | 5,353,344 | 4,799,857 |
| | Genome coverage (%) | 79.9 | 65.3 | 79.8 | 76.8 | 82.0 | 84.5 | 70.4 | 67.5 | 68.3 | 47.3 | 65.8 | 68.9 |
| | NGA50 (bp) | 27,878 | 17,230 | 12,950 | 16,314 | 34,687 | 6,075 | 163 | 743 | 3,020 | 126 | 367 | 429 |
| | NGA75 (bp) | 7,625 | 1,000 | 3,219 | 1,897 | 9,956 | 861 | 163 | 163 | 141 | 75 | 256 | 264 |
| | **MGA scaffolds** | | | | | | | | | | | | |
| | Number | 1,274 | 182 | 906 | 255 | 518 | 4,260 | 13,023 | 5,481 | 2,719 | 1,636 | 5,724 | 6,973 |
| | Size (bp) | 5,392,598 | 3,888,252 | 6,514,949 | 3,993,851 | 8,586,377 | 3,693,139 | 4,780,266 | 4,569,851 | 4,491,934 | 3,149,938 | 4,288,561 | 4,384,528 |
| | Genome coverage (%) | **80.9** | **71.9** | **80.3** | **76.8** | **82.6** | **81.4** | **73.2** | **68.4** | **73.7** | **57.9** | **68.9** | **72.5** |
| | NGA50 (bp) | 65,397 | 38,226 | 35,910 | 23,434 | 85,203 | 8,447 | 1,834 | 3,884 | 11,298 | 4,168 | 2,138 | 1,487 |
| | NGA75 (bp) | 35,351 | 13,154 | 28,846 | 13,075 | 69,382 | 3,702 | 556 | 2,110 | 4,191 | 1483 | 906 | 739 |
| | | | | | | | | | | | | | |
| | Increased NGA50 compared with TGB | 121.56 | 749.53 | 1.68 | 459.49 | 2.24 | 13.65 | 2.04 | 12.45 | 45.74 | 81.73 | 6.58 | 3.22 |
| | Increased NGA75 compared with TGB | 693.16 | 257.92 | 17.04 | 256.37 | 7.59 | 72.59 | 10.90 | 41.37 | 82.18 | 29.08 | 12.08 | 4.65 |
| | Increased NGA50 compared with contigs | 2.35 | 2.22 | 2.77 | 1.44 | 2.46 | 1.39 | 11.25 | 5.23 | 3.74 | 33.08 | 5.83 | 3.47 |
| | increased NGA75 compared with contigs | 4.64 | 13.15 | 8.96 | 6.89 | 6.97 | 4.30 | 3.41 | 12.94 | 29.72 | 19.77 | 3.54 | 2.80 |
| | Meta-O coverage (%) | 86 | 75 | 81 | 77 | 88 | 88 | 84 | 74 | 84 | 70 | 77 | 76 |
| | Enhanced coverage (%) | 25.4 | 24.6 | 3.9 | 36.0 | 0.0 | 20.6 | 5.4 | 15.4 | 22.7 | 16.8 | 14.4 | 4.5 |
| | Relative recovery ratio (%) | 0.94 | 0.96 | 0.99 | 0.99 | 0.94 | 0.93 | 0.87 | 0.93 | 0.88 | 0.83 | 0.90 | 0.96 |
| | | | | | | | | | | | | | |
| metaBAT | Genome coverage (%) | **73.67** | **72.83** | **57.462** | **63.337** | **65.67** | **39.05** | **NA** | **NA** | **2.346** | **NA** | **NA** | **NA** |
| | NGA50 (bp) | 26,144 | 33,876 | 6,047 | 11,334 | 23,437 | NA | NA | NA | NA | NA | NA | NA |
| | NGA75 (bp) | 17,511 | NA | NA | NA | NA | NA | NA | NA | NA | NA | NA | NA |

## Supplementary Table 7. Statistics of the assembled kelp bacterial genomes.

| Genome id | Assembly size | Completeness (%) | Contamination (%) | Scaffold number | Average scaffold length | Scaffold N50 | Maximum scaffold length |
|---|---|---|---|---|---|---|---|
| 014 | 3,828,953 | 100 | 1.49 | 160 | 23,930 | 414,263 | 1,106,013 |
| 168 | 7,370,036 | 100 | 1.11 | 208 | 35,432 | 933,744 | 1,337,279 |
| 022 | 3,782,646 | 100 | 0.81 | 96 | 39,402 | 220,806 | 973,932 |
| 121 | 7,419,534 | 100 | 1.11 | 82 | 90,482 | 1,013,440 | 1,315,040 |
| 132 | 4,618,743 | 99.66 | 0.79 | 262 | 17,628 | 476,846 | 873,507 |
| 47 | 4,525,403 | 99.5 | 0.5 | 57 | 79,393 | 903,837 | 2,150,235 |
| 105 | 5,911,000 | 99.35 | 1.23 | 510 | 11,590 | 38,492 | 144,670 |
| 90 | 5,296,229 | 99.15 | 3.51 | 448 | 11,821 | 44,614 | 157,902 |
| 56 | 3,581,442 | 99.13 | 1.14 | 138 | 25,952 | 519,557 | 796,115 |
| 153 | 3,811,156 | 99.13 | 4.55 | 61 | 62,477 | 520,228 | 1,033,196 |
| 10 | 3,990,084 | 98.84 | 1.16 | 92 | 43,370 | 727,735 | 892,637 |
| 12 | 2,945,076 | 98.81 | 3.08 | 243 | 12,119 | 45,581 | 111,607 |
| 421 | 5,785,613 | 98.62 | 7.8 | 103 | 56,171 | 743,283 | 1,597,741 |
| 77 | 5,547,643 | 98.62 | 1.27 | 46 | 120,600 | 632,653 | 1,651,731 |
| 3 | 5,544,724 | 98.43 | 0.55 | 12 | 349,360 | 1,241,311 | 1,499,708 |
| 111 | 4,913,560 | 98.18 | 0.66 | 296 | 16,599 | 45,788 | 211,625 |
| 117 | 10,851,072 | 97.98 | 2.99 | 756 | 14,353 | 61,845 | 226,400 |
| 55 | 5,529,155 | 97.44 | 2.14 | 108 | 51,195 | 1,241,179 | 1,834,826 |
| 201 | 6,501,654 | 97.2 | 9.43 | 577 | 11,268 | 87,950 | 213,484 |
| 235 | 5,111,833 | 96.96 | 0.43 | 105 | 48,684 | 170,651 | 417,177 |
| 89 | 5,405,063 | 96.85 | 1.49 | 124 | 43,589 | 237,828 | 439,892 |
| 178 | 8,224,003 | 96.73 | 3.72 | 193 | 42,611 | 716,450 | 1,098,200 |
| 158 | 5,905,942 | 96.55 | 8.62 | 98 | 60,264 | 392,896 | 736,722 |
| 41 | 5,100,233 | 96.55 | 1.15 | 63 | 80,956 | 272,347 | 806,318 |
| 87 | 2,957,218 | 96.11 | 0.37 | 33 | 89,612 | 745,817 | 1,457,745 |
| 130 | 3,088,588 | 95.75 | 3.07 | 1,424 | 2,168 | 321,316 | 534,710 |
| 29 | 5,092,361 | 95.4 | 2.3 | 99 | 51,437 | 184,212 | 596,877 |
| 165 | 4,424,455 | 95.29 | 0.51 | 48 | 92,176 | 873,504 | 1,364,475 |
| 51 | 7,878,100 | 94.32 | 1.56 | 298 | 26,436 | 102,504 | 453,014 |
| 112 | 5,287,582 | 94.25 | 9.2 | 433 | 12,211 | 118,045 | 413,164 |
| 151 | 5,927,155 | 94.15 | 7.33 | 1,209 | 4,902 | 78,380 | 248,805 |
| 11 | 7,326,264 | 93.1 | 1.42 | 1,801 | 4,067 | 105,733 | 831,318 |

| | | | | | | | |
|---|---|---|---|---|---|---|---|
| 113 | 4,671,280 | 93.1 | 0.32 | 114 | 40,976 | 92,184 | 295,298 |
| 1 | 4,392,158 | 91.23 | 10.25 | 78 | 56,309 | 446,149 | 1,196,081 |
| 73 | 5,739,693 | 91.05 | 3.84 | 1,140 | 5,034 | 972,735 | 1,978,628 |
| 52 | 3,631,852 | 90.55 | 0 | 96 | 37,831 | 906,266 | 1,370,565 |
| 95 | 4,285,344 | 88.35 | 10.83 | 205 | 20,904 | 141,659 | 621,247 |
| 20 | 3,581,647 | 87.96 | 3.68 | 2,344 | 1,528 | 996,230 | 1,694,935 |
| 72 | 6,240,548 | 86.36 | 0 | 104 | 60,005 | 496,645 | 1,105,389 |
| 118 | 6,078,849 | 85.55 | 3.2 | 176 | 34,538 | 146,938 | 359,242 |
| 115 | 6,001,581 | 84.66 | 4.55 | 47 | 127,693 | 385,648 | 1,105,304 |
| 27 | 5,583,057 | 84.48 | 0.79 | 1,163 | 4,800 | 72,819 | 247,657 |
| 160 | 3,529,502 | 83.58 | 0 | 31 | 113,854 | 2,277,313 | 2,277,313 |
| 38 | 8,366,548 | 82.07 | 5.62 | 2,085 | 4,012 | 8,767 | 66,301 |
| 108 | 3,823,737 | 81.91 | 2.14 | 129 | 29,641 | 59,578 | 147,327 |
| 162 | 4,936,729 | 81.66 | 3.17 | 858 | 5,753 | 601,317 | 1,329,838 |
| 68 | 2,893,920 | 81.63 | 0 | 836 | 3,461 | 12,543 | 51,941 |
| 142 | 4,729,776 | 79.31 | 4.36 | 1,017 | 4,650 | 9,830 | 76,204 |
| 49 | 3,852,396 | 78.45 | 3.64 | 1,872 | 2,057 | 6,060 | 170,067 |
| 143 | 4,858,525 | 77.67 | 2.42 | 3,110 | 1,562 | 15,813 | 89,333 |
| 56 | 4,463,152 | 77.59 | 2.93 | 1,518 | 2,940 | 76,494 | 300,064 |
| 154 | 6,828,745 | 77.35 | 0 | 379 | 18,017 | 34,556 | 212,760 |
| 5 | 2,400,131 | 76.81 | 2.05 | 146 | 16,439 | 22,251 | 72,590 |
| 150 | 8,538,199 | 74.39 | 1.42 | 613 | 13,928 | 36,783 | 213,494 |
| 171 | 6,100,915 | 71.25 | 0 | 329 | 18,543 | 25,010 | 98,022 |
| 176 | 5,527,469 | 70.84 | 7.78 | 675 | 8,188 | 239,387 | 1,337,462 |
| 34 | 3,775,652 | 69.83 | 0 | 111 | 34,014 | 61,099 | 165,897 |
| 181 | 5,286,309 | 68.97 | 0 | 251 | 21,060 | 93,704 | 584,148 |
| 46 | 3,794,245 | 67.55 | 6.18 | 573 | 6,621 | 17,722 | 78,608 |
| 59 | 4,066,146 | 67.22 | 3.11 | 522 | 7,789 | 12,190 | 48,349 |
| 129 | 1,971,621 | 63.07 | 1.18 | 491 | 4,015 | 6,425 | 28,848 |
| 166 | 3,776,634 | 59.86 | 1.85 | 2,262 | 1,669 | 1,957 | 406,721 |
| 91 | 6,994,949 | 54.8 | 2.04 | 1,191 | 5,873 | 537,997 | 1,074,938 |
| 35 | 3,073,698 | 54.59 | 5 | 461 | 6,667 | 12,381 | 41,472 |
| 134 | 4,000,118 | 52.66 | 2.55 | 1,863 | 2,147 | 5,212 | 272,239 |
| 119 | 2,448,852 | 50 | 0 | 171 | 14,320 | 1,499,652 | 1,499,652 |
| 71 | 4,332,981 | 34.48 | 0 | 538 | 8,053 | 367,194 | 1,076,623 |
| 76 | 2,693,622 | 28.43 | 0 | 754 | 3,572 | 4,462 | 20,107 |

| | | | | | | | |
|---|---|---|---|---|---|---|---|
| 135 | 1,451,267 | 25 | 0 | 472 | 3,074 | 3,679 | 12,393 |
| 6 | 357,154 | 19.32 | 0 | 89 | 4,012 | 16,798 | 36,941 |
| 159 | 568,943 | 18.52 | 0 | 169 | 3,366 | 12,275 | 28,825 |
| 92 | 1,624,168 | 17.24 | 0 | 348 | 4,667 | 7,158 | 54,357 |
| 137 | 488,892 | 14.93 | 0 | 8 | 61,111 | 484,413 | 484,413 |
| 42 | 243,383 | 12.07 | 0 | 120 | 2,028 | 2,146 | 17,756 |
| 172 | 1,580,725 | 11.01 | 0 | 801 | 1,973 | 2,157 | 8,622 |

## Supplementary Table 8. Enriched KEGG functions in the assembled kelp bacterial genomes compared with gut and ocean microbiomes.

| Description | Enriched pathway | ES (kelp vs ocean) | ES (kelp vs gut) |
|---|---|---|---|
| Overview | Carbon metabolism [PATH:ko01200] | 6,071 | 9,471 |
| | 2-Oxocarboxylic acid metabolism [PATH:ko01210] | 1,028 | 1,742 |
| | Fatty acid metabolism [PATH:ko01212] | 1,670 | 1,985 |
| | Biosynthesis of amino acids [PATH:ko01230] | 7,067 | 13,052 |
| Carbohydrate metabolism | Glycolysis / Gluconeogenesis [PATH:ko00010] | 2,353 | 3,039 |
| | Citrate cycle (TCA cycle) [PATH:ko00020] | 1,538 | 2,328 |
| | Pentose phosphate pathway [PATH:ko00030] | 1,718 | 3,253 |
| | Pentose and glucuronate interconversions [PATH:ko00040] | 684 | 847 |
| | Fructose and mannose metabolism [PATH:ko00051] | 1,068 | 1,863 |
| | Galactose metabolism [PATH:ko00052] | 483 | 641 |
| | Ascorbate and aldarate metabolism [PATH:ko00053] | 375 | 564 |
| | Starch and sucrose metabolism [PATH:ko00500] | 1,470 | 2,058 |
| | Pyruvate metabolism [PATH:ko00620] | 2,843 | 4,080 |
| | Glyoxylate and dicarboxylate metabolism [PATH:ko00630] | 2,624 | 4,659 |
| | Propanoate metabolism [PATH:ko00640] | 1,838 | 2,398 |
| | Butanoate metabolism [PATH:ko00650] | 2,502 | 2,875 |
| | Inositol phosphate metabolism [PATH:ko00562] | 327 | 408 |
| Energy metabolism | Oxidative phosphorylation [PATH:ko00190] | 1,586 | 3,551 |
| | Photosynthesis [PATH:ko00195] | 352 | 1,120 |
| | Photosynthesis - antenna proteins [PATH:ko00196] | 19 | 36 |
| | Carbon fixation in photosynthetic organisms | 912 | 2,188 |

| | | | |
|---|---|---|---|
| | [PATH:ko00710] | | |
| | Carbon fixation pathways in prokaryotes [PATH:ko00720] | 1,748 | 2,654 |
| | Methane metabolism [PATH:ko00680] | 1,649 | 2,423 |
| | Nitrogen metabolism [PATH:ko00910] | 1,215 | 1,958 |
| metabolism | Fatty acid biosynthesis [PATH:ko00061] | 983 | 1,275 |
| | Fatty acid elongation [PATH:ko00062] | 41 | 46 |
| | Fatty acid degradation [PATH:ko00071] | 1,855 | 1,778 |
| | Synthesis and degradation of ketone bodies [PATH:ko00072] | 746 | 579 |
| | Cutin, suberine and wax biosynthesis [PATH:ko00073] | 4 | 4 |
| | Steroid biosynthesis [PATH:ko00100] | 108 | 119 |
| | Primary bile acid biosynthesis [PATH:ko00120] | 84 | 115 |
| | Steroid hormone biosynthesis [PATH:ko00140] | 62 | 75 |
| | Glycerolipid metabolism [PATH:ko00561] | 428 | 624 |
| | Glycerophospholipid metabolism [PATH:ko00564] | 951 | 1,469 |
| | Ether lipid metabolism [PATH:ko00565] | 137 | 125 |
| | Sphingolipid metabolism [PATH:ko00600] | 133 | 138 |
| | Linoleic acid metabolism [PATH:ko00591] | 54 | 73 |
| | alpha-Linolenic acid metabolism [PATH:ko00592] | 215 | 179 |
| | Biosynthesis of unsaturated fatty acids [PATH:ko01040] | 334 | 362 |
| Nucleotide metabolism | Purine metabolism [PATH:ko00230] | 2,178 | 5,428 |
| | Pyrimidine metabolism [PATH:ko00240] | 1,363 | 3,263 |
| acid metabolism | Alanine, aspartate and glutamate metabolism [PATH:ko00250] | 1,545 | 2,837 |
| | Glycine, serine and threonine metabolism [PATH:ko00260] | 3,477 | 5,567 |
| | Cysteine and methionine metabolism [PATH:ko00270] | 1,861 | 3,607 |
| | Valine, leucine and isoleucine biosynthesis [PATH:ko00290] | 1,197 | 1,265 |
| | Lysine biosynthesis [PATH:ko00300] | 597 | 1,232 |
| | Lysine degradation [PATH:ko00310] | 545 | 386 |
| | Arginine biosynthesis [PATH:ko00220] | 948 | 1,627 |
| | Arginine and proline metabolism [PATH:ko00330] | 1,687 | 2,690 |
| | Histidine metabolism [PATH:ko00340] | 1,454 | 2,067 |
| | Tyrosine metabolism [PATH:ko00350] | 1,128 | 1,329 |

| | | | |
|---|---|---|---|
| | Tryptophan metabolism [PATH:ko00380] | 841 | 1,199 |
| | Phenylalanine, tyrosine and tryptophan biosynthesis [PATH:ko00400] | 846 | 1,968 |
| Metabolism of other amino acids | Taurine and hypotaurine metabolism [PATH:ko00430] | 456 | 355 |
| | Phosphonate and phosphinate metabolism [PATH:ko00440] | 155 | 236 |
| | Selenocompound metabolism [PATH:ko00450] | 768 | 1,069 |
| | Cyanoamino acid metabolism [PATH:ko00460] | 146 | 166 |
| | D-Glutamine and D-glutamate metabolism [PATH:ko00471] | 237 | 548 |
| | D-Arginine and D-ornithine metabolism [PATH:ko00472] | 28 | 70 |
| | D-Alanine metabolism [PATH:ko00473] | 112 | 274 |
| | Glutathione metabolism [PATH:ko00480] | 781 | 1,446 |
| Glycan biosynthesis and metabolism | N-Glycan biosynthesis [PATH:ko00510] | 7 | 14 |
| | Other types of O-glycan biosynthesis [PATH:ko00514] | 23 | 7 |
| | Glycosaminoglycan biosynthesis - heparan sulfate / heparin [PATH:ko00534] | 15 | 15 |
| | Glycosaminoglycan degradation [PATH:ko00531] | 730 | 552 |
| | Glycosphingolipid biosynthesis - lacto and neolacto series [PATH:ko00601] | 17 | 14 |
| | Lipopolysaccharide biosynthesis [PATH:ko00540] | 550 | 2,014 |
| | Peptidoglycan biosynthesis [PATH:ko00550] | 671 | 1,764 |
| Metabolism of cofactors and vitamins | Riboflavin metabolism [PATH:ko00740] | 414 | 787 |
| | Vitamin B6 metabolism [PATH:ko00750] | 697 | 1,142 |
| | Nicotinate and nicotinamide metabolism [PATH:ko00760] | 809 | 1,483 |
| | Pantothenate and CoA biosynthesis [PATH:ko00770] | 1,114 | 1,862 |
| | Biotin metabolism [PATH:ko00780] | 708 | 797 |
| | Lipoic acid metabolism [PATH:ko00785] | 121 | 134 |
| | Folate biosynthesis [PATH:ko00790] | 695 | 1,512 |
| | One carbon pool by folate [PATH:ko00670] | 577 | 1,288 |
| | Porphyrin and chlorophyll metabolism [PATH:ko00860] | 2,288 | 4,393 |
| | Ubiquinone and other terpenoid-quinone biosynthesis [PATH:ko00130] | 1,324 | 2,209 |
| Metabolism of terpenoids and polyketides | Terpenoid backbone biosynthesis [PATH:ko00900] | 552 | 1,135 |

| | | | |
|---|---|---|---|
| | Carotenoid biosynthesis [PATH:ko00906] | 179 | 355 |
| | Insect hormone biosynthesis [PATH:ko00981] | 6 | 6 |
| | Limonene and pinene degradation [PATH:ko00903] | 448 | 247 |
| | Geraniol degradation [PATH:ko00281] | 537 | 441 |
| | Tetracycline biosynthesis [PATH:ko00253] | 64 | 160 |
| | Polyketide sugar unit biosynthesis [PATH:ko00523] | 16 | 59 |
| | Nonribosomal peptide structures [PATH:ko01054] | 61 | 86 |
| | Biosynthesis of siderophore group nonribosomal peptides [PATH:ko01053] | 142 | 154 |
| | Biosynthesis of vancomycin group antibiotics [PATH:ko01055] | 0 | 4 |
| Biosynthesis of other secondary metabolites | Phenylpropanoid biosynthesis [PATH:ko00940] | 61 | 141 |
| | Isoquinoline alkaloid biosynthesis [PATH:ko00950] | 73 | 103 |
| | Tropane, piperidine and pyridine alkaloid biosynthesis [PATH:ko00960] | 104 | 102 |
| | Betalain biosynthesis [PATH:ko00965] | 66 | 91 |
| | Penicillin and cephalosporin biosynthesis [PATH:ko00311] | 53 | 106 |
| | Carbapenem biosynthesis [PATH:ko00332] | 15 | 41 |
| | Monobactam biosynthesis [PATH:ko00261] | 223 | 382 |
| | Streptomycin biosynthesis [PATH:ko00521] | 372 | 702 |
| | Novobiocin biosynthesis [PATH:ko00401] | 80 | 90 |
| Xenobiotics biodegradation and metabolism | Aminobenzoate degradation [PATH:ko00627] | 575 | 856 |
| | Fluorobenzoate degradation [PATH:ko00364] | 304 | 358 |
| | Chloroalkane and chloroalkene degradation [PATH:ko00625] | 589 | 623 |
| | Toluene degradation [PATH:ko00623] | 21 | 11 |
| | Nitrotoluene degradation [PATH:ko00633] | 346 | 246 |
| | Caprolactam degradation [PATH:ko00930] | 297 | 387 |
| | Bisphenol degradation [PATH:ko00363] | 243 | 116 |
| | Dioxin degradation [PATH:ko00621] | 51 | 118 |
| | Steroid degradation [PATH:ko00984] | 1,044 | 1,043 |
| | Metabolism of xenobiotics by cytochrome P450 [PATH:ko00980] | 143 | 110 |
| | Drug metabolism - cytochrome P450 [PATH:ko00982] | 221 | 199 |
| Transcription | RNA polymerase [PATH:ko03020] | 8 | 80 |
| Translation | Ribosome [PATH:ko03010] | 1,101 | 2,647 |

| | | | |
|---|---|---|---|
| | Aminoacyl-tRNA biosynthesis [PATH:ko00970] | 834 | 2,546 |
| | Ribosome biogenesis in eukaryotes [PATH:ko03008] | 10 | 20 |
| Folding, sorting and degradation | Protein export [PATH:ko03060] | 345 | 891 |
| | Ubiquitin mediated proteolysis [PATH:ko04120] | 10 | 10 |
| | Sulfur system [PATH:ko04122] | 580 | 1,163 |
| | Proteasome [PATH:ko03050] | 6 | 11 |
| | RNA degradation [PATH:ko03018] | 453 | 709 |
| Replication and repair | Base excision repair [PATH:ko03410] | 477 | 946 |
| | Nucleotide excision repair [PATH:ko03420] | 96 | 331 |
| Membrane transport | ABC transporters [PATH:ko02010] | 7,850 | 10,495 |
| | Phosphotransferase system (PTS) [PATH:ko02060] | 52 | 76 |
| | Bacterial secretion system [PATH:ko03070] | 1,094 | 1,719 |
| Signal transduction | Two-component system [PATH:ko02020] | 7,556 | 9,257 |
| | Rap1 signaling pathway [PATH:ko04015] | 4 | 3 |
| | MAPK signaling pathway [PATH:ko04010] | 4 | 4 |
| | ErbB signaling pathway [PATH:ko04012] | 3 | 3 |
| | Hedgehog signaling pathway [PATH:ko04340] | 9 | 8 |
| | Hippo signaling pathway [PATH:ko04390] | 3 | 3 |
| | Hippo signaling pathway -fly [PATH:ko04391] | 11 | 10 |
| | VEGF signaling pathway [PATH:ko04370] | 5 | 5 |
| | TNF signaling pathway [PATH:ko04668] | 1 | 1 |
| | HIF-1 signaling pathway [PATH:ko04066] | 60 | 60 |
| | FoxO signaling pathway [PATH:ko04068] | 20 | 31 |
| | Calcium signaling pathway [PATH:ko04020] | 22 | 24 |
| | Phosphatidylinositol signaling system [PATH:ko04070] | 42 | 53 |
| | Sphingolipid signaling pathway [PATH:ko04071] | 15 | 20 |
| | cAMP signaling pathway [PATH:ko04024] | 23 | 24 |
| | cGMP - PKG signaling pathway [PATH:ko04022] | 29 | 30 |
| | Plant hormone signal transduction [PATH:ko04075] | 5 | 5 |
| Singling molecules and interaction | ECM-receptor interaction [PATH:ko04512] | 26 | 36 |
| Transport and catabolism | Phagosome [PATH:ko04145] | 7 | 9 |
| | Lysosome [PATH:ko04142] | 261 | 210 |
| Cell motility | Bacterial chemotaxis [PATH:ko02030] | 1,166 | 1,657 |

| | | | |
|---|---|---|---|
| Cell growth and death | Cell cycle - Caulobacter [PATH:ko04112] | 1,259 | 2,311 |
| | Meiosis - yeast [PATH:ko04113] | 12 | 9 |
| | Oocyte meiosis [PATH:ko04114] | 10 | 10 |
| | Apoptosis [PATH:ko04210] | 5 | 2 |
| | p53 signaling pathway [PATH:ko04115] | 5 | 2 |
| Cellular commiunity | Focal adhesion [PATH:ko04510] | 15 | 19 |
| | Tight junction [PATH:ko04530] | 7 | 6 |
| | Cell adhesion molecules [PATH:ko045140] | 3 | 35 |
| | Gap junction [PATH:ko04540] | 14 | 13 |
| Immune system | Hematopoietic cell lineage [PATH:ko04640] | 3 | 3 |
| | Platelet activation [PATH:ko04611] | 14 | 15 |
| | Leukocyte transendothelial migration [PATH:ko04670] | 1 | 1 |
| | Chemokine signaling pathway [PATH:ko04062] | 2 | 2 |
| Endocrine system | Insulin secretion [PATH:ko04911] | 9 | 9 |
| | Glucagon signaling pathway [PATH:ko04922] | 135 | 155 |
| | Regulation of lipolysis in adipocyte [PATH:ko04923] | 18 | 21 |
| | GnRH signaling pathway [PATH:ko04912] | 15 | 16 |
| | Oxytocin signaling pathway [PATH:ko04921] | 32 | 33 |
| | Thyroid hormone signaling pathway [PATH:ko04919] | 6 | 5 |
| | Melanogenesis [PATH:ko04916] | 7 | 8 |
| | Renin secretion [PATH:ko04924] | 20 | 21 |
| | Renin-angiotensin system [PATH:ko04614] | 10 | 20 |
| | Aldosterone synthesis and secretion [PATH:ko04925] | 35 | 36 |
| Circulatory system | Cardiac muscle contraction [PATH:ko04260] | 21 | 88 |
| | Adrenergic signaling in cardiomyocytes [PATH:ko04261] | 11 | 11 |
| | Vascular smooth muscle contraction [PATH:ko04270] | 26 | 27 |
| Digestive system | Salivary secretion [PATH:ko04970] | 17 | 18 |
| | Gastric acid secretion [PATH:ko04971] | 5 | 6 |
| | Pancreatic secretion [PATH:ko04972] | 14 | 14 |
| | Fat digestion and absorption [PATH:ko04975] | 2 | 1 |
| | Vitamin digestion and absorption [PATH:ko04977] | 1 | 1 |
| Development | Circadian entrainment [PATH:ko04713] | 18 | 19 |
| Environmental adaptation | Plant-pathogen interaction [PATH:ko04626] | 64 | 129 |

**Supplementary Table 9. Detection of CAZyme related HGTs from the assembled kelp bacterial genomes.**

| | EGGNOG annotation | NR annotation | Taxonomic annotation |
|---|---|---|---|
| HD029 | | | Bacteria; Planctomycetes; Planctomycetia; Pirellulales;Pirellulaceae |
| PROKKA 02066 | succinoglycan biosynthesis | succinoglycan biosynthesis ketolase | Bacteria; Proteobacteria; Gammaproteobacteria; Xanthomonadales; Xanthomonadaceae; Rhodanobacter; Rhodanobacter thiooxydans |
| PROKKA 02067 | glycosyl transferase group 1 | glycosyl transferase group 1 | Bacteria; Proteobacteria; delta/epsilon subdivisions; Deltaproteobacteria; Desulfovibrionales; Desulfohalobiaceae; Desulfonatronospira; Desulfonatronospira thiodismutans |
| PROKKA 02068 | succinoglycan biosynthesis | succinoglycan biosynthesis ketolase | Bacteria; Proteobacteria; Gammaproteobacteria; Xanthomonadales; Xanthomonadaceae; Rhodanobacter; Rhodanobacter thiooxydans |
| PROKKA 02069 | ABC transporter | polysaccharide polyol phosphate abc transporter atp-binding protein | Bacteria; Planctomycetes; Planctomycetia; Planctomycetales; Planctomycetaceae; Rhodopirellula |
| | | | |
| HD041 | | | Bacteria; Planctomycetes; Planctomycetia; Pirellulales;Pirellulaceae |
| PROKKA 01728 | abc-2 type transporter | abc-2 type transporter | Bacteria; Planctomycetes; Planctomycetia; Planctomycetales; Planctomycetaceae; Rhodopirellula |

| | | | |
|---|---|---|---|
| PROKKA 01729 | ABC transporter | polysaccharide polyol phosphate abc transporter atp-binding protein | Bacteria; Planctomycetes; Planctomycetia; Planctomycetales; Planctomycetaceae; Rhodopirellula |
| PROKKA 01730 | succinoglycan biosynthesis | succinoglycan biosynthesis ketolase | Bacteria; Proteobacteria; Gammaproteobacteria; Xanthomonadales; Xanthomonadaceae; Rhodanobacter; Rhodanobacter thiooxydans |
| PROKKA 01731 | glycosyl transferase group 1 | glycosyl transferase group 1 | Bacteria; Proteobacteria; delta/epsilon subdivisions; Deltaproteobacteria; Desulfovibrionales; Desulfohalobiaceae; Desulfonatronospira; Desulfonatronospira thiodismutans |
| PROKKA 01732 | succinoglycan biosynthesis | succinoglycan biosynthesis ketolase | Bacteria; Proteobacteria; Gammaproteobacteria; Xanthomonadales; Xanthomonadaceae; Rhodanobacter; Rhodanobacter thiooxydans |
| | | | |
| HD056 | | | Bacteria; Proteobacteria; Gammaproteobacteria; Alteromonadales; Alteromonadaceae; Marinobacter |
| PROKKA 01908 | Glycosyl transferase (Group 1 | glycosyltransferase | Bacteria; Proteobacteria; Gammaproteobacteria; Alteromonadales; Alteromonadaceae; Marinobacter; |
| PROKKA 01909 | Polysaccharide Biosynthesis Protein | virulence factor mvin superfamily | Bacteria; Proteobacteria; Gammaproteobacteria; Alteromonadales; Alteromonadaceae; Marinobacter; |

| | | | |
|---|---|---|---|
| PROKKA 01910 | pyruvyl transferase | colanic acid biosynthesis glycosyl transferase | Bacteria; Proteobacteria; Gammaproteobacteria; Alteromonadales; Alteromonadaceae; Marinobacter; |
| PROKKA 01911 | Sulfotransferase domain | sulfotransferase domain | Bacteria; Proteobacteria; Gammaproteobacteria; Alteromonadales; Alteromonadaceae; Marinobacter; |
| PROKKA 01912 | glycosyl transferase group 1 | glycosyltransferase involved in cell wall bisynthesis | Bacteria; Proteobacteria; Gammaproteobacteria; Alteromonadales; Alteromonadaceae; Marinobacter; |
| PROKKA 01913 | Glycosyl transferase (Group 1 | glycosyltransferase involved in cell wall bisynthesis | Bacteria; Proteobacteria; Gammaproteobacteria; Alteromonadales; Alteromonadaceae; Marinobacter; |
| | | | |
| HD001 | | | Bacteria; Proteobacteria; Gammaproteobacteria |
| PROKKA 02788 | integral membrane protein | rnd transporter | Bacteria; Proteobacteria; Gammaproteobacteria; Oceanospirillales; Alcanivoracaceae; Alcanivorax |
| PROKKA 02789 | pump that utilizes the energy of pyrophosphate hydrolysis as the driving force for | multispecies: pyrophosphatase | Bacteria; Proteobacteria; Alphaproteobacteria; Rhodobacterales; Rhodobacteraceae; Pannonibacter; Pannonibacter phragmitetus |
| PROKKA 02790 | Major Facilitator superfamily | mfs transporter | Bacteria; Proteobacteria; Alphaproteobacteria; Rhodobacterales; Rhodobacteraceae; Roseibium |

| | | | |
|---|---|---|---|
| PROKKA 02791 | Catalyzes the ATP-dependent phosphorylation of thiamine-monophosphate (TMP) to form thiamine-pyrophosphate (TPP), the active form of vitamin B1 (By similarity) | thiamine-monophosphate kinase | Bacteria; Proteobacteria; Alphaproteobacteria; Rhizobiales; Rhodobiaceae; Amorphus; Amorphus coralli |
| PROKKA 02792 | Involved in the transcription termination process (By similarity) | antitermination protein | Bacteria; Proteobacteria; Alphaproteobacteria; Rhizobiales; Rhodobiaceae; Amorphus; Amorphus coralli |
| | | | |
| HD056 | | | |
| PROKKA 03652 | no hit | membrane hypothetical protein | Bacteria; Proteobacteria; Gammaproteobacteria; Enterobacteriales; Enterobacteriaceae; Xenorhabdus; Xenorhabdus nematophila |
| PROKKA 03653 | no hit | asparagine synthase | Bacteria; Proteobacteria; Gammaproteobacteria; Vibrionales; Vibrionaceae; Vibrio; Vibrio cholerae |
| PROKKA 03654 | no hit | glycosyltransferase | Bacteria; Proteobacteria; Gammaproteobacteria; Vibrionales; Vibrionaceae; Vibrio; Vibrio cholerae |
| | | | |
| HD091 | | | Bacteria; Bacteroidetes/Chlorobi group; Bacteroidetes; Flavobacteriia; |
| PROKKA 03207 | LPS biosynthesis protein WbpG | lps biosynthesis protein | Bacteria; Proteobacteria; Betaproteobacteria; Burkholderiales; Alcaligenaceae; Achromobacter |
| PROKKA 03208 | Polysaccharide biosynthesis protein | polysaccharide biosynthesis protein | Bacteria; Bacteroidetes/Chlorobi group; Bacteroidetes; Cytophagia; Cytophagales; Cytophagaceae; Pontibacter |

| | | | |
|---|---|---|---|
| PROKKA 03209 | Polysaccharide biosynthesis protein | polysaccharide biosynthesis protein | Bacteria; Bacteroidetes/Chlorobi group; Bacteroidetes; Flavobacteriia; Flavobacteriales; Flavobacteriaceae; Aequorivita; Aequorivita sublithincola |
| | | | |
| HD073 | | | Bacteria; Proteobacteria; Gammaproteobacteria; |
| PROKKA 03560 | Inherit from NOG: protein N-acetylglucosaminyltransferase activity | hypothetical protein | Bacteria; Proteobacteria; delta/epsilon subdivisions; Deltaproteobacteria; Syntrophobacterales; Syntrophaceae; Desulfomonile; Desulfomonile tiedjei |
| PROKKA 03561 | Glucose-1-phosphate cytidylyltransferase | glucose-1-phosphate cytidylyltransferase | Archaea; Euryarchaeota; Methanomicrobia; Methanosarcinales; Methanosarcinaceae; Methanococcoides; Methanococcoides burtonii |
| PROKKA 03562 | Glycosyl transferase | glycosyl hydrolase | Bacteria; Proteobacteria; Alphaproteobacteria; Rhodobacterales; Rhodobacteraceae; Rhodovulum |
| PROKKA 03563 | GtrA-like protein | polysaccharide biosynthesis protein | Bacteria; Proteobacteria; Zetaproteobacteria; Mariprofundales; Mariprofundaceae; Mariprofundus; Mariprofundus ferrooxydans |
| PROKKA 03564 | Nad-dependent epimerase dehydratase | gdp-6-deoxy-d-lyxo-4-hexulose reductase | Bacteria; Proteobacteria; Alphaproteobacteria; Rhizobiales; Bradyrhizobiaceae; Bradyrhizobium |

| | | | |
|---|---|---|---|
| PROKKA 03565 | Sulfotransferase | antigen biosynthesis sulfotransferas | Bacteria; Armatimonadetes; Chthonomonadetes; Chthonomonadales; Chthonomonadaceae; Chthonomonas; Chthonomonas calidirosea |
| HD073 | | | Bacteria; Proteobacteria; Gammaproteobacteria; |
| PROKKA 03567 | Transketolase | transketolase c-terminal section | Bacteria; Proteobacteria; Alphaproteobacteria; Rhizobiales; Bradyrhizobiaceae; Bradyrhizobium |
| PROKKA 03568 | Transketolase | transketolase subunit a | Bacteria; Proteobacteria; Alphaproteobacteria; Rhizobiales; Bradyrhizobiaceae; Bradyrhizobium |
| PROKKA 03569 | cDP-glucose 4,6-dehydratase | cdp-glucose 4%2c6-dehydratase | Bacteria; Proteobacteria; Gammaproteobacteria; Enterobacteriales; Enterobacteriaceae; Yersinia; Yersinia pseudotuberculosis complex; Yersinia similis |
| | | | |
| HD134 | | | Bacteria; Proteobacteria; Alphaproteobacteria; Rhodospirillales; Rhodospirillaceae |
| PROKKA 01963 | Gluconate | gluconate transporter | Bacteria; Planctomycetes; Planctomycetia; Planctomycetales; Planctomycetaceae; Gimesia; Gimesia maris |
| PROKKA 01964 | rotein of unknown function (DUF1549) | probable vegetatible incompatibility protein het-e-1 | Bacteria; Planctomycetes; Planctomycetia; Planctomycetales; Planctomycetaceae; Gimesia; Gimesia maris |

| | | | |
|---|---|---|---|
| PROKKA 01965 | rotein of unknown function (DUF1549) | hypothetical protein | Bacteria; Planctomycetes; Planctomycetia; Planctomycetales; Planctomycetaceae; Gimesia; Gimesia maris |
| PROKKA 01966 | 4-alpha-glucanotransferase (EC 2.4.1.25) | alpha-amylase | Bacteria; Planctomycetes; Planctomycetia; Planctomycetales; Planctomycetaceae; Gimesia; Gimesia maris |
| PROKKA 01967 | Galactose-1-phosphate uridyl transferase, N-terminal domain | galactose-1-phosphate uridylyltransferase | Bacteria; Planctomycetes; Planctomycetia; Planctomycetales; Planctomycetaceae; Gimesia; Gimesia maris |
| PROKKA 01968 | Synthesizes alpha-1,4-glucan chains using ADP-glucose | glycogen synthase | Bacteria; Planctomycetes; Planctomycetia; Planctomycetales; Planctomycetaceae; Gimesia; Gimesia maris |
| PROKKA 01969 | Signal transduction histidine kinase | two-component system sensor histidine kinase | Bacteria; Planctomycetes; Planctomycetia; Planctomycetales; Planctomycetaceae; Gimesia; Gimesia maris |
| PROKKA 01970 | two component, sigma54 specific, transcriptional regulator, | transcriptional regulator | Bacteria; Planctomycetes; Planctomycetia; Planctomycetales; Planctomycetaceae; Gimesia; Gimesia maris |
| PROKKA 01971 | Capsular exopolysaccharide family | capsular exopolysaccharide family protein | Bacteria; Planctomycetes; Planctomycetia; Planctomycetales; Planctomycetaceae; Gimesia; Gimesia maris |
| PROKKA 01972 | Nad-dependent epimerase dehydratase | vi polysaccharide biosynthesis protein | Bacteria; Planctomycetes; Planctomycetia; Planctomycetales; Planctomycetaceae; Gimesia; Gimesia maris |

## Supplementary Table 10. Enriched EGGNOG functions of HGTs.

| EGGNOG id | q value | Function annotation |
|---|---|---|
| ENOG4108JTR | 0.000218332733213567 | (EAL) domain protein |
| ENOG410689M | 3.15717204881535e-05 | (LipO)protein |
| ENOG4105W0E | 6.1765344220446e-31 | 50S ribosomal protein L33 |
| ENOG41086UE | 0.00339447313191844 | 50S ribosomal protein l34 |
| ENOG41082BR | 0.000166204976004298 | 50S ribosomal protein L35 |
| ENOG4108VRW | 0.00339447313191844 | ABC transporter, ATP-binding protein-related protein |
| ENOG41090SZ | 2.82032025016974e-06 | ABC-2 type transporter |
| ENOG4105N02 | 0.00156965762943297 | ABC-type amino acid transport signal transduction |
| ENOG4105HXF | 0.00072699345714977 | accessory protein |
| ENOG4105PSZ | 0.00156965762943297 | Activator of Hsp90 ATPase 1 family protein |
| ENOG4106AYV | 0.00855314566638699 | Acyl carrier protein |
| ENOG4105X59 | 0.00565706457669922 | Addiction Module Antidote |
| ENOG4105DVP | 0.00855314566638699 | Adenine-specific |
| ENOG4108IFH | 0.00339447313191844 | alkyl hydroperoxide reductase Thiol specific antioxidant Mal allergen |
| ENOG4105ZSN | 0.00072699345714977 | amidohydrolase |
| ENOG4106Q5N | 0.00616645743569505 | amidohydrolase 2 |
| ENOG4105R5T | 1.55099462266912e-05 | Antitoxin of toxin-antitoxin stability system |
| ENOG4105D1M | 0.0011836748909562 | ATP-dependent endonuclease of the OLD |
| ENOG4107B22 | 5.49439973385136e-06 | Bacterial aa3 type cytochrome c oxidase subunit IV |
| ENOG4106ECH | 0.00339447313191844 | Bacteriophage CI repressor helix-turn-helix domain |
| ENOG4106SJ0 | 0.00855314566638699 | Binding-protein-dependent transport system inner membrane component |
| ENOG4108PEE | 0.00855314566638699 | binding-protein-dependent transport systems inner membrane Component |
| ENOG4107FH8 | 0.00339447313191844 | Biopolymer transport protein exbD tolR |
| ENOG4106191 | 0.00944375999158841 | bleomycin resistance protein |
| ENOG4105W16 | 0.000644690500477891 | bluf domain protein |
| ENOG41076SS | 0.00279475600532592 | BON domain |
| ENOG4106R79 | 7.72543651763318e-07 | C4-dicarboxylate transporter family protein DctM subunit |
| ENOG4106VMP | 0.00855314566638699 | CAAX protease self-immunity |
| ENOG41068WT | 0.00339447313191844 | Calcineurin-like phosphoesterase |
| ENOG4108W0M | 0.00156965762943297 | calcium-binding |
| ENOG4105VNB | 1.85051816351537e-10 | Carrier of the growing fatty acid chain in fatty acid biosynthesis (By similarity) |

| | | |
|---|---|---|
| ENOG4108Z6K | 0.00279475600532592 | Catalyzes the circularization of gamma-N-acetyl-alpha,gamma-diaminobutyric acid (ADABA) to ectoine (1,4,5,6-tetrahydro-2-methyl-4-pyrimidine carboxylic acid), which is an excellent osmoprotectant (By similarity) |
| ENOG4107BV8 | 0.000218332733213567 | Cfr10I/Bse634I restriction endonuclease |
| ENOG4107452 | 0.00855314566638699 | Chromosome segregation ATPase |
| ENOG4105X2W | 0.00279475600532592 | Coenzyme PQQ synthesis protein D (PqqD) |
| ENOG4105VEQ | 2.07190860137704e-05 | Cold shock protein |
| ENOG410864Z | 0.000218332733213567 | conserved small protein containing a coiled-coil domain |
| ENOG410861M | 0.00944375999158841 | Csbd family |
| ENOG4106BUR | 0.00156965762943297 | Curli production assembly/transport component CsgG |
| ENOG4107YVV | 0.00072699345714977 | Cytidyltransferase-related domain protein |
| ENOG4106GGE | 0.000218332733213567 | cytochrome oxidase subunit |
| ENOG4105NI8 | 0.000315107251835354 | Cytosine-specific methyltransferase |
| ENOG4107U4U | 0.00339447313191844 | DEAD DEAH box helicase |
| ENOG4107S3V | 0.00339447313191844 | deaminase |
| ENOG4105SN0 | 0.00339447313191844 | DegT/DnrJ/EryC1/StrS aminotransferase family |
| ENOG4106JJJ | 0.000218332733213567 | DNA helicase |
| ENOG4107V4W | 0.00339447313191844 | DNA Methylase |
| ENOG4108223 | 0.00072699345714977 | dna mismatch repair |
| ENOG4108R3I | 0.00072699345714977 | dna polymerase iii |
| ENOG41082JX | 0.00038778798291652 | Excinuclease ABC C subunit domain protein |
| ENOG4107U0R | 0.00339447313191844 | Excinuclease ATPase subunit |
| ENOG4105I71 | 0.00339447313191844 | extracellular solute-binding protein family 1 |
| ENOG4107R3N | 0.000218332733213567 | Filamentation induced by cAMP protein fic |
| ENOG4105XK1 | 1.55099462266912e-05 | flagellar biosynthesis regulatory protein FlaF |
| ENOG4105TXQ | 0.00855314566638699 | Flagellar hook-length control protein FliK |
| ENOG4107KI8 | 0.00339447313191844 | Flp/Fap pilin component |
| ENOG4105HAQ | 0.000218332733213567 | Frg domain protein |
| ENOG4105MJM | 0.000218332733213567 | FRG domain-containing protein |
| ENOG4105EJZ | 0.000218332733213567 | Glucose-1-phosphate cytidylyltransferase |
| ENOG4108YMS | 0.00339447313191844 | glycosyl transferase family |
| ENOG4107Y14 | 0.0078568848458097 | Glycosyl transferase, family 2 |
| ENOG4105H0D | 0.00072699345714977 | GntR Family Transcriptional Regulator |
| ENOG4107ZHZ | 7.72543651763318e-07 | group 1 glycosyl transferase |
| ENOG4108N2K | 0.00339447313191844 | Heat shock protein |
| ENOG4105W3S | 3.63654907791715e-05 | hemerythrin-like, metal-binding protein |

| | | |
|---|---|---|
| ENOG41061PY | 0.00339447313191844 | Histidine kinase |
| ENOG4105R50 | 0.00339447313191844 | Hnh endonuclease |
| ENOG4105K9U | 4.32371322564174e-13 | however, it seems to stimulate more or less all the activities of the other two initiation factors, IF-2 and IF-3 (By similarity) |
| ENOG4108WS3 | 0.000218332733213567 | HTTM domain protein |
| ENOG4108YSZ | 0.00339447313191844 | Inherit from COG: Archaeal ATPase |
| ENOG4105H1W | 0.00855314566638699 | Inherit from COG: Ferredoxin |
| ENOG41081N3 | 0.000218332733213567 | Inherit from COG: helicase |
| ENOG4105Q9P | 0.00393242317393556 | Inherit from COG: UPF0754 membrane protein |
| ENOG4108JAX | 0.00339447313191844 | Inherit from COG: wd repeat |
| ENOG41085Q6 | 1.55099462266912e-05 | Inherit from NOG: Pentapeptide repeat-containing protein |
| ENOG4107NB2 | 2.82032025016974e-06 | Inherit from NOG: Xis-like protein |
| ENOG41081J2 | 0.000160144088714481 | integral membrane protein |
| ENOG4105VTR | 0.00246299685368339 | Integrase |
| ENOG4108JFP | 0.00339447313191844 | Integrase core domain |
| ENOG41083TA | 0.00855314566638699 | Integrase, catalytic region |
| ENOG4105M10 | 0.00944375999158841 | Involved in the anomeric conversion of L-rhamnose (By similarity) |
| ENOG4106C1H | 0.000218332733213567 | Involved in the biosynthesis of D-alanyl-lipoteichoic acid (LTA). Activated D-alanyl-Dcp donates its D-alanyl substituent to membrane-associated LTA (By similarity) |
| ENOG4105KEN | 0.000166204976004298 | Involved in the modulation of the specificity of the ClpAP-mediated ATP-dependent protein degradation (By similarity) |
| ENOG4108UQY | 0.000157294853262952 | Iron-storage protein |
| ENOG4105JTJ | 0.00339447313191844 | Isochorismatase, hydrolase |
| ENOG4105M43 | 0.00156965762943297 | isoprenylcysteine carboxyl methyltransferase |
| ENOG4105DD7 | 0.00339447313191844 | kinase (PfkB family |
| ENOG4105UYT | 0.00944375999158841 | limonene-12-epoxide hydrolase |
| ENOG41082S2 | 0.000218332733213567 | lipolytic protein G-D-S-L family |
| ENOG4107W19 | 0.00393242317393556 | lmbE family |
| ENOG4107J8N | 0.00339447313191844 | LPS biosynthesis protein WbpG |
| ENOG4108MW0 | 0.00855314566638699 | Major Facilitator |
| ENOG4106VY7 | 0.00339447313191844 | MAPEG family |
| ENOG4108BKE | 0.00279475600532592 | Membrane |
| ENOG4106DXD | 0.00339447313191844 | metal-binding protein |
| ENOG4105EUX | 0.000783745012153436 | methane phenol toluene hydroxylase |
| ENOG4108V0U | 0.000218332733213567 | Methyl-accepting chemotaxis sensory transducer |

| | | |
|---|---|---|
| ENOG4105D85 | 0.000133443410650813 | methylase |
| ENOG4106AH5 | 7.72543651763318e-07 | Mitochondrial domain of unknown function (DUF1713) |
| ENOG4108MCH | 0.00339447313191844 | Modifies the free amino group of the aminoacyl moiety of methionyl-tRNA(fMet). The formyl group appears to play a dual role in the initiator identity of N-formylmethionyl-tRNA by (I) promoting its recognition by IF2 and (II) impairing its binding to EFTu-GTP (By similarity) |
| ENOG4108ZFP | 0.000218332733213567 | monooxygenase component MmoB DmpM |
| ENOG4105E56 | 0.00339447313191844 | MT-A70 family |
| ENOG4105QV3 | 0.00339447313191844 | MTH538 TIR-like domain (DUF1863) |
| ENOG4107YT8 | 0.00339447313191844 | n-acetylmuramoyl-l-alanine amidase |
| ENOG4105KWU | 2.03925201704501e-08 | NDH-1 shuttles electrons from NADH, via FMN and iron- sulfur (Fe-S) centers, to quinones in the respiratory chain |
| ENOG4105M5H | 6.34402090118427e-05 | NDH-1 shuttles electrons from NADH, via FMN and iron- sulfur (Fe-S) centers, to quinones in the respiratory chain. The immediate electron acceptor for the enzyme in this species is believed to be ubiquinone. Couples the redox reaction to proton translocation (for every two electrons transferred, four hydrogen ions are translocated across the cytoplasmic membrane), and thus conserves the redox energy in a proton gradient (By similarity) |
| ENOG4105IDK | 1.05597418390806e-05 | Nitrogen fixation protein of unknown function |
| ENOG4105NWT | 0.00855314566638699 | Nuclear protein set |
| ENOG4108BJE | 0.000218332733213567 | o-methyltransferase family 3 |
| ENOG4107A2S | 0.00339447313191844 | Orthopoxvirus protein of unknown function (DUF830) |
| ENOG4108HMS | 1.55099462266912e-05 | Oxidoreductase FAD-binding domain |
| ENOG4105JPY | 0.00339447313191844 | ParB domain protein nuclease |
| ENOG410861S | 0.000166204976004298 | Part of the twin-arginine translocation (Tat) system that transports large folded proteins containing a characteristic twin-arginine motif in their signal peptide across membranes. TatA could form the protein-conducting channel of the Tat system (By similarity) |
| ENOG41060WK | 0.000157294853262952 | PAS domain |
| ENOG4107H1E | 0.000218332733213567 | PBS lyase HEAT domain protein repeat-containing protein |
| ENOG4105MYW | 0.000783745012153436 | Pentapeptide repeat protein |
| ENOG4107P08 | 0.00565706457669922 | PEP-CTERM motif |
| ENOG4105CTG | 0.00855314566638699 | peptidase S8 and S53, subtilisin, kexin, sedolisin |
| ENOG4108IN1 | 0.00339447313191844 | peptide Chain Release factor |
| ENOG4108KTA | 0.000218332733213567 | Periplasmic protein-like protein |
| ENOG4105ERM | 0.000315107251835354 | Periplasmic serine protease |
| ENOG4107USW | 0.00339447313191844 | pfkb domain protein |

| | | |
|---|---|---|
| ENOG4108RA6 | 0.00072699345714977 | phage Tail Protein |
| ENOG4107RCJ | 0.00393242317393556 | Phage Tail Tape Measure Protein |
| ENOG4106ESZ | 0.00565706457669922 | Phasin protein |
| ENOG4105VE1 | 0.00339447313191844 | Phosphatidylcholine synthase |
| ENOG4105XTZ | 0.00339447313191844 | PilT protein domain protein |
| ENOG41078VE | 0.00339447313191844 | PIN domain |
| ENOG4105KBS | 0.00855314566638699 | Plasmid maintenance system killer |
| ENOG4106DD1 | 0.000218332733213567 | Plasmid stabilization system |
| ENOG4108VQB | 0.000218332733213567 | Polysaccharide biosynthesis protein |
| ENOG4107I1A | 0.000218332733213567 | Polysaccharide pyruvyl transferase |
| ENOG4106IM3 | 0.00339447313191844 | Predicted small integral membrane protein (DUF2160) |
| ENOG41088AN | 0.00855314566638699 | Preprotein translocase |
| ENOG4105Y3P | 4.36079233952542e-08 | Preprotein translocase SecE subunit |
| ENOG41086AV | 0.000160144088714481 | Preprotein translocase subunit SecG |
| ENOG4107U70 | 0.00339447313191844 | PTS system sorbose-specific iic component |
| ENOG4106R6M | 0.00339447313191844 | Putative papain-like cysteine peptidase (DUF1796) |
| ENOG4108MBF | 0.00110454355958953 | Pyrrolo-quinoline quinone |
| ENOG41085PJ | 0.000218332733213567 | rdd domain containing protein |
| ENOG4108AQK | 0.00339447313191844 | Redoxin |
| ENOG41090XY | 0.000218332733213567 | Redoxin domain protein |
| ENOG4105PM9 | 0.00855314566638699 | repressor |
| ENOG4107TJZ | 0.00855314566638699 | resolvase |
| ENOG4105QC3 | 0.00156965762943297 | response regulator |
| ENOG4105QB9 | 0.00339447313191844 | response regulator receiver protein |
| ENOG4105PCJ | 0.00339447313191844 | Restriction endonuclease EcoRI |
| ENOG41090ME | 1.05597418390806e-05 | Restriction modification system DNA specificity |
| ENOG410801J | 0.000218332733213567 | reverse transcriptase |
| ENOG4108SK8 | 0.00339447313191844 | Reverse transcriptase (RNA-dependent DNA polymerase) |
| ENOG41073XN | 0.00339447313191844 | Ribosomal RNA methyltransferase (FmrO) |
| ENOG4105KW8 | 4.26367205997395e-07 | RNA chaperone that binds small regulatory RNA (sRNAs) and mRNAs to facilitate mRNA translational regulation in response to envelope stress, environmental stress and changes in metabolite concentrations. Also binds with high specificity to tRNAs (By similarity) |
| ENOG4105SPA | 0.000218332733213567 | RNA Polymerase |
| ENOG4106439 | 0.00565706457669922 | s-layer domain-containing protein |
| ENOG4106DBW | 0.000546580503981913 | SAP domain |

| | | |
|---|---|---|
| ENOG4108XZN | 0.00855314566638699 | SARP family transcriptional regulator |
| ENOG4107S7F | 0.00339447313191844 | Secreted protein |
| ENOG41081WR | 0.00339447313191844 | Sel1 repeat |
| ENOG41083W0 | 7.72543651763318e-07 | septation inhibitor protein |
| ENOG4106AEM | 0.00393242317393556 | sigma-54 modulation protein |
| ENOG4108VU7 | 0.00339447313191844 | sigma-70 region 2 |
| ENOG4105ZBM | 0.00156965762943297 | Small integral membrane protein |
| ENOG4105ZFI | 0.00339447313191844 | SMI1 / KNR4 family (SUKH-1) |
| ENOG4105NW6 | 1.55099462266912e-05 | solute symporter protein |
| ENOG4108XXH | 0.00072699345714977 | specificity |
| ENOG4105T84 | 0.00072699345714977 | Sporulation domain-containing protein |
| ENOG410682B | 0.00279475600532592 | stage II sporulation protein |
| ENOG4108RT4 | 3.39219647387324e-08 | succinoglycan biosynthesis |
| ENOG4108T2N | 0.00279475600532592 | Sulfopyruvate decarboxylase |
| ENOG4108X99 | 0.00221548560276533 | Sulfotransferase domain |
| ENOG4108SD0 | 6.45507457431872e-05 | Sulfotransferase family |
| ENOG4105TAT | 0.00565706457669922 | Sulfur oxidation protein |
| ENOG4107UN7 | 0.00339447313191844 | T5orf172 |
| ENOG4105C2K | 0.0078568848458097 | tail SHEATH protein |
| ENOG4108WH5 | 0.00565706457669922 | TetR family Transcriptional regulator |
| ENOG410850R | 0.00339447313191844 | Tetratricopeptide TPR 2 repeat protein |
| ENOG410826X | 3.63654907791715e-05 | This protein binds to 23S rRNA in the presence of protein L20 (By similarity) |
| ENOG4105KJA | 0.000218332733213567 | Toxic component of a toxin-antitoxin (TA) module. A |
| ENOG4105DW1 | 0.000166204976004298 | Transcriptional regulator, ARAC family |
| ENOG41068IM | 0.00156965762943297 | transcriptional regulator, copG family |
| ENOG4108T13 | 0.000315107251835354 | Transcriptional regulator, Crp Fnr family |
| ENOG4108V5Q | 0.00156965762943297 | transglutaminase-like protein |
| ENOG4105XAK | 0.00339447313191844 | transglycosylase associated protein |
| ENOG4108XII | 0.00279475600532592 | Transporter DctQ |
| ENOG4108K6H | 0.00156965762943297 | transposase |
| ENOG4106AGW | 0.00339447313191844 | Tripartite ATP-independent periplasmic transporters, DctQ component |
| ENOG410696S | 1.55099462266912e-05 | Tryptophan-rich protein (DUF2389) |
| ENOG4106GNM | 0.00339447313191844 | Two component transcriptional regulator (Winged helix family |
| ENOG4105EZ1 | 0.00279475600532592 | type I restriction enzyme |
| ENOG4108WC9 | 0.00072699345714977 | type VI secretion system effector, hcp1 family |

| | | |
|---|---|---|
| ENOG4105VP3 | 1.99535676508832e-07 | Uncharacterized protein conserved in bacteria (DUF2191) |
| ENOG4107F20 | 7.72543651763318e-07 | UPF0391 membrane protein |
| ENOG4105P51 | 0.00279475600532592 | Usg family |
| ENOG41075PN | 0.00339447313191844 | VanZ like family |
| ENOG4105ECI | 0.000783745012153436 | YeeC-like protein |
| ENOG4105VPI | 0.000218332733213567 | YqaE family transport protein |
| ENOG4105GY0 | 0.000218332733213567 | Zeta toxin |
